# Periodic acid-promoted methylenation of imidazoheteroarenes: a green approach using ethylene glycol as a C1 source

**DOI:** 10.1039/d5ra09947a

**Published:** 2026-03-02

**Authors:** Marcelo S. Franco, Matheus Y. G. Watanabe, Jhefferson S. Guilhermi, Brunno S. Souza, Sumbal Saba, Jamal Rafique, Antonio L. Braga

**Affiliations:** a Departamento de Química, Universidade Federal de Santa Catarina Florianópolis 88040-900 Santa Catarina Brazil braga.antonio@ufsc.br; b Laboratory of Sustainable Synthesis and Organochalcogen (LabSO), Instituto de Química, Universidade Federal de Goiás – UFG Goiânia 74690-900 Goiás Brazil sumbalsaba@ufg.br; c Instituto de Química, Universidade Federal do Mato Grosso do Sul – UFMS Campo Grande 79074-460 Mato Grosso do Sul Brazil jamal.rafique@ufms.br

## Abstract

Herein, we describe an environmentally sustainable methodology for the methylenation of C3(sp^2^) imidazoheteroarenes, employing formaldehyde (C1) generated *in situ via* oxidative cleavage of ethylene glycol—a renewable feedstock. The protocol utilizes water as a non-toxic solvent and stoichiometric reagents, aligning with green chemistry principles by ensuring high atomic economy and minimal waste. Optimized conditions enable regioselective bis-heterocycle formation with yields up to 97%. The reaction proceeds through a Malaprade oxidation-mediated ionic pathway mediated by 0.5 molar equiv. of periodic acid, and demonstrates broad substrate scope, including imidazo[1,2-*a*]pyridines and imidazo[2,1-*b*]thiazoles. Scalability was validated (70% yield at 5.5 mmol scale), highlighting its potential for industrial applications. This method offers a robust, metal-free alternative for C–H functionalization.

## Abstract 2

Periodic acid promotes metal-free methylenation of imidazoheteroarenes using ethylene glycol as a green C1 source. The reaction proceeds in water, delivers yields up to 97%, & is scalable, offering a sustainable approach to bis-heterocycle synthesis.

## Graphical abstract

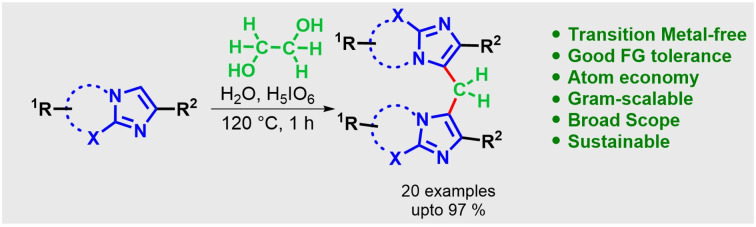

Nitrogen-containing heterocycles represent a major field of study in organic chemistry.^1^ Research in this area is extensive, driven by the significant physiological and pharmacological properties of N-heterocyclic compounds.^2,3^ They serve as fundamental building blocks for many biologically important molecules, including vitamins, nucleic acids, and pharmaceuticals.^1–3^ Their broad applicability stems from their ability to act as proton donors or acceptors and to engage in diverse non-covalent interactions, such as hydrogen bonding, van der Waals forces, hydrophobic effects, and π-stacking.^4^

Vitaku, Smith, and Njardarson (2014) published a comprehensive analysis of the structural diversity, substitution patterns, and frequency of nitrogen-containing heterocycles in US FDA-approved drugs. Their work highlights the relevance of these structures, which range from active components in therapeutic agents to key building blocks for new drug candidates.^5^

In recent decades, numerous methods have been developed for the construction and functionalization of imidazoheterocycles.^6^ Among these, imidazo[1,2-*a*]pyridine (IP) is particularly prominent. This scaffold is present in many commercial drugs, such as Zolpidem®, Necopidem®, Zolimidine®, and Minodronic acid® (Fig. 1), making it an important core structure with a wide range of structural variations.^7^ Furthermore, IP derivatives possess significant luminescent properties,^8–12^ and are also employed as fluorescent sensors for metal ions,^9,13^ and in phosphorescent complexes for light-emitting devices.^14^

**Fig. 1:**
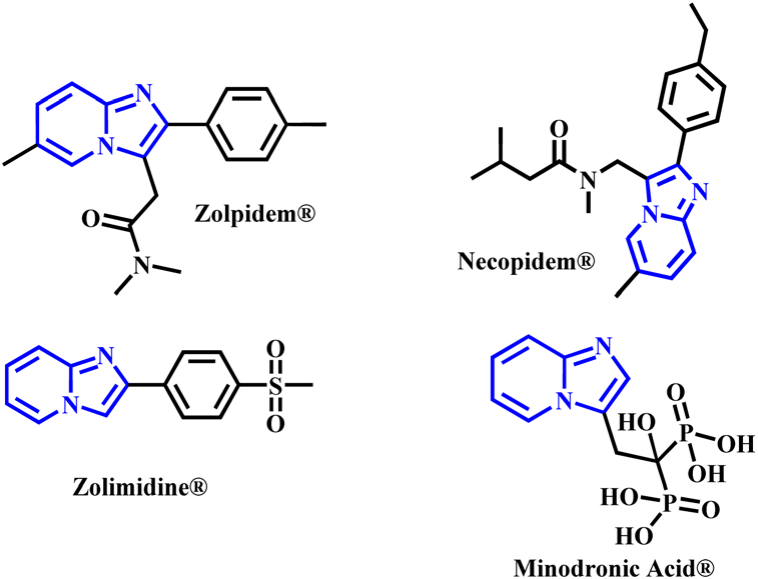
Imidazo[1,2-*a*]pyridine (IP) containing commercialized drugs.

In organic synthesis, ethylene glycol is known as a valuable two-carbon (C2) synthon but it can also be strategically cleaved to function as a C1 source for the construction of various one-carbon functional groups. Under appropriate conditions, the C–C bond of ethylene glycol can be cleaved, to generate formaldehyde *in situ*.^15^ The released formaldehyde can then be trapped *in situ* to form methylene bridges or incorporate into heterocycles.^16^

Over recent decades, several research groups have developed methods for the functionalization of the C–H bonds in IPs. Most of these methods focus on the nucleophilic C-3 position. The ability to achieve this selective functionalization without needing to protect the C-3 site is particularly advantageous from a synthetic perspective.^7^ Consequently, one important functionalization of IPs is methylenation (Scheme 1). The insertion of a methylene group into a molecule is a highly attractive synthetic strategy for constructing complex structures, especially bis-heterocyclic systems.^17^

**Scheme 1:**
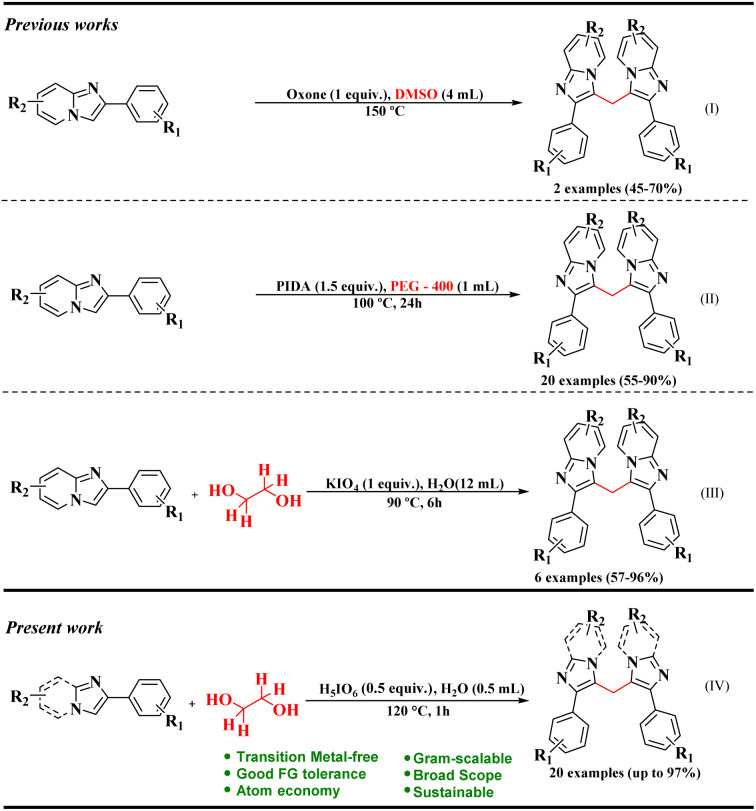
Methylenation reactions in literature.

As part of our research interest in designing and developing sustainable processes, as well as the C(sp^2^)–H functionalization of biologically-relevant heteroarenes,^18,19^ herein, we describe, a regioselective and environmentally benign protocol for the methylenation *via* Malaprade oxidation. Based on the interest in creating new, more sustainable synthetic routes for IP derivatives.

In the first part of the new protocol, studies were carried out to optimize the reaction conditions using 2-phenylimidazo[1,2-*a*]pyridine(1a) (0.3 mmol), ethylene glycol (2a) (1 molar equivalent) and 0.5 mL of distilled water. As expected, the reaction does not occur without the presence of an oxidizing agent, for 1 h at 100 °C (Table 1, entry 1). Therefore, an attempt was made to find an oxidizing agent, testing iodic acid (HIO_3_), sodium iodide (NaIO_3_) and potassium iodide (KIO_3_), keeping the other conditions constant. However, reactions did not occur (entries 2, 3 and 4, respectively). The addition of periodic acid proved to be efficient as an oxidizing agent for the system, obtaining 73% of the 2-phenylimidazo[1,2-*a*]pyridine dimer (3a) and 24% of the hydroxymethylated IP (4a) (entry 5).

**Table 1:** Optimization of reaction conditions^a^

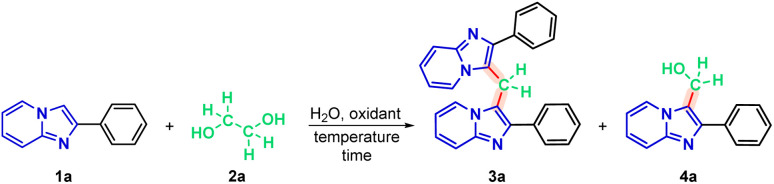						
Entry	2a (equiv.)	Oxidant (equiv.)	Temperature (°C)	Time (h)	Yield^b^ (%)	
					3a	4a
1	1	−	100	1	—	—
2	1	HIO_3_ (1)	100	1	—	—
3	1	NaIO_3_ (1)	100	1	—	—
4	1	KIO_3_ (1)	100	1	—	—
5	1	H_5_IO_6_ (1)	100	1	73	24
6	0.5	H_5_IO_6_ (0.5)	100	1	79	7
7	0.4	H_5_IO_6_ (0.4)	100	1	69	8
8	0.5	H_5_IO_6_ (0.5)	110	1	88	5
9	0.5	H_5_IO_6_ (0.5)	120	1	95	n.d.
10	0.5	H_5_IO_6_ (0.5)	120	0.5	62	14

a Conditions: 1a (0.3 mmol), 2a (molar equivalent), oxidant (molar equivalent), H_2_O (0.5 mL), time (hour), temperature (°C). —: no reaction. −: absence. n.d.: not detected.

b Isolated yield.

In order to increase the yield of the dimer, a reduction of 1 molar equivalent to 0.5 of reagent 2a was performed to make it more chemoselective, proving to be efficient, since the yield was increased from 73% to 79% of the desired product (3a) and, reduced from 24% to 7% of the by-product (4a) (entry 6). When the reduction was performed to 0.4 molar equivalent of 2a, a reduction to 69% of the dimer (3a) and to 8% of the alcohol of 2-phenylimidazo[1,2-*a*]pyridine (4a) was observed (entry 7).

The next modification performed was the temperature, testing at 110 °C and 120 °C, obtaining, respectively, excellent values of 88% for the dimer (3a) and 5% for the alcohol (4a) (entry 8); and only the dimer (3a) in 95% yield (entry 9).

Finally, we tried to optimize the reaction time, reducing it to 30 min (entry 10), which proved to be inefficient compared to the best condition, entry 9, since with the shortest time, 62% of the desired dimer (3a) and 14% of the hydroxymethylated by-product (4a).

With the optimal reaction conditions established (Scheme 2), we applied the optimized methodology to a series of substrates derived from IPs to evaluate the generality of the new protocol.

**Scheme 2:**
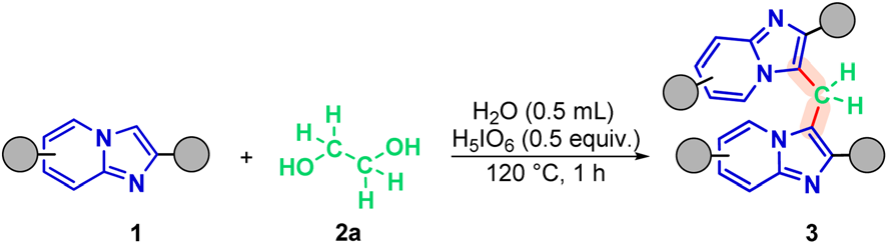
Optimized protocol of methylenation of imidazo[1,2-*a*]pyridine 1. Conditions: 1 (0.3 mmol), 2a (0.5 molar equivalent), H_5_IO_6_ (0.5 equiv.), H_2_O (0.5 mL), time (1 h), temperature (120 °C).

When donating groups were attached to the pyridine nucleus of the 2-phenylimidazo[1,2-*a*]pyridine scaffold, the corresponding products (3b–3d, 3n) were isolated in excellent yields of 76–97%. A similar monosubstituted system with an electron-withdrawing group (3i) was also synthesized, albeit in a lower yield of 53% (Fig. 2).

**Fig. 2:**
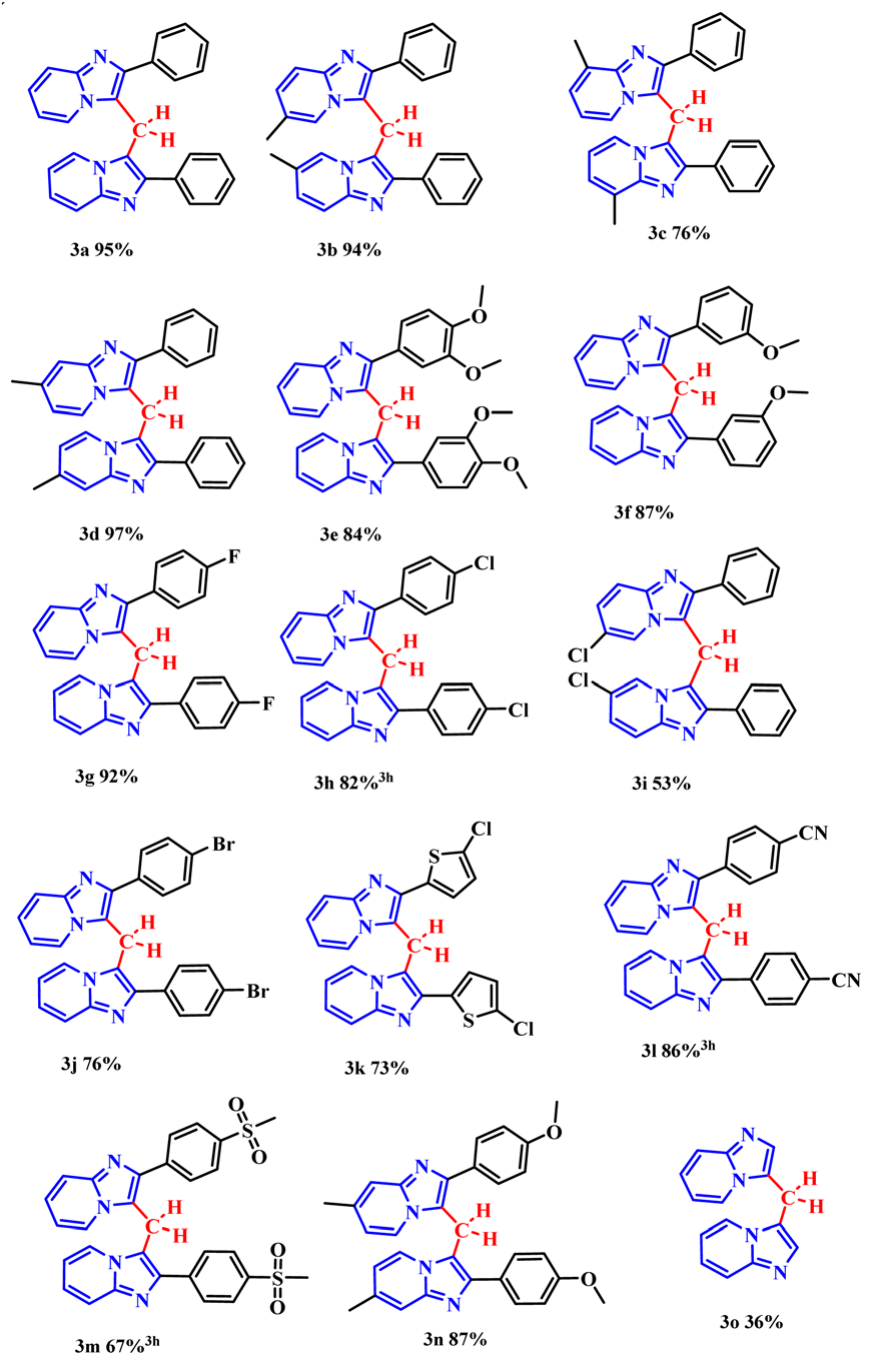
Scope of imidazo[1,2-*a*]pyridine.

The influence of substituents on the pendant phenyl group was also investigated. Electron-donating groups (1e, 1f) provided high yields (84–87%) of 3e and 3f. Conversely, electron-withdrawing groups (1g, 1h, 1j–1o) resulted in a broader yield range of 55–87% for products 3g, 3h, 3j–3n (Fig. 2). The reaction of the unsubstituted IP precursor proceeded with low efficiency, yielding only 36% of 3o. This lower yield could be due to the lack of the stabilizing aryl substituent that is present in all other substrates (3a–3n).

To prove the versatility of the method, the protocol was applied to other N-heteroarene nuclei, such as imidazo[2,1-*b*]thiazole 1A (Scheme 3).

**Scheme 3:**
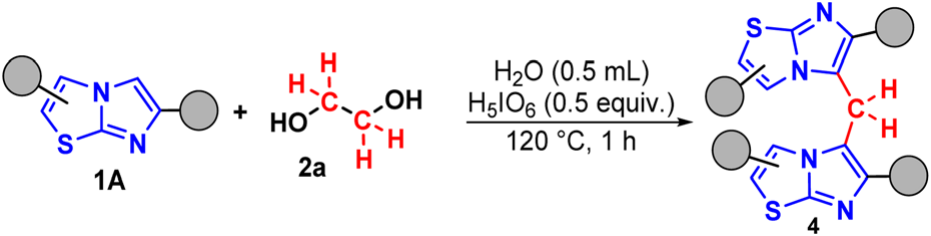
Optimized protocol of methylenation of imidazo[2,1-*b*]thiazole 1A. Conditions: 1A (0.3 mmol), 2a (0.5 molar equivalent), H_5_IO_6_ (0.5 equiv.), H_2_O (0.5 mL), time (1 h), temperature (120 °C).

In general, the desired products 4a–d were obtained in good to excellent yields. When evaluating the compounds, the influence of electronic effects is notable. Compounds with electron-withdrawing groups linked to the phenyl ring, such as 4d and 4e, presented yields in the range of 58–63%. In contrast, the compound with an electron-donating group, 4c, presented a significantly higher yield of 89% (Fig. 3).

**Fig. 3:**
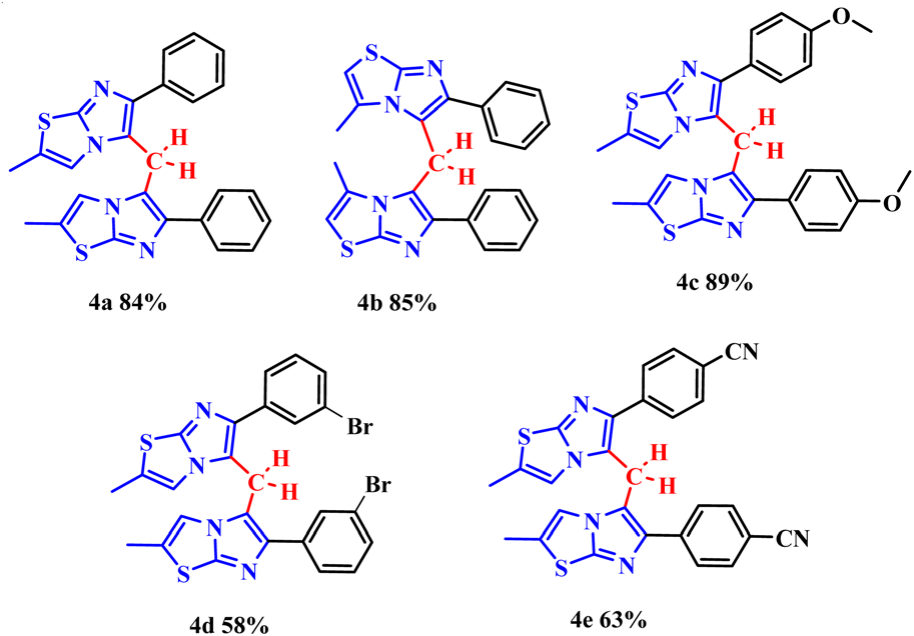
Scope of imidazo[2,1-*b*]thiazoles.

To evaluate the method's variability compared to other sources of methylene groups, reactions were performed using glycerol (2b) and PEG-400 (2c). The reaction with glycerol gave a 92% yield, while the one with PEG-400 gave only 8% (Table 2).

**Table 2:** Methylene group source experiments^a^

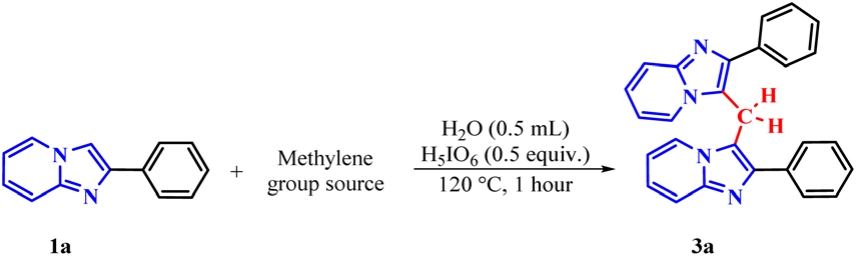		
Entry	Methylene group source	3a, yield^b^ (%)
1	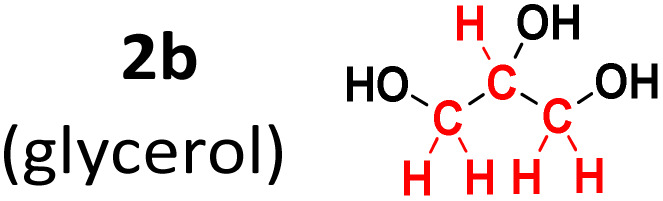	93%
2	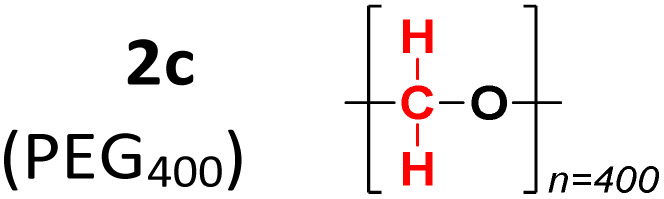	8%

a Conditions: 1a (0.3 mmol), 2 (molar equivalent), H_5_IO_6_ (0.5 molar equivalent), H_2_O (0.5 mL), time (1 h), temperature (120 °C).

b Isolated yield.

In the next step, a scale-up experiment was performed using 5.5 mmol of 1a, ethylene glycol (2.75 mmol, 0.5 equiv.), and periodic acid (H_5_IO_6_, 2.75 mmol, 0.5 equiv.) in water (10 mL). The reaction mixture was heated at 120 °C for 6 hours, as a longer reaction time was anticipated to be necessary for the increased scale. The desired product (3a) was obtained in 70% yield (0.7738 g), demonstrating that the method can accommodate variations in scale.

In order to elucidate the mechanism by which the dimer formation reaction takes place, the mechanism of the methylenation reaction was examined. By adding TEMPO to the standard experiment conditions, product 3a was obtained in 92% yield (Table 3, entry 1), suggesting that the reaction occurs *via* an ionic pathway. The experiment was also carried out under an argon atmosphere (Table 3, entry 2), obtaining 93% yield, verifying that O_2_ does not have an active role in the reaction mechanism.

**Table 3:** Control experiments^a^

Entry	Control experiments	3a, yield^b^ (%)
1	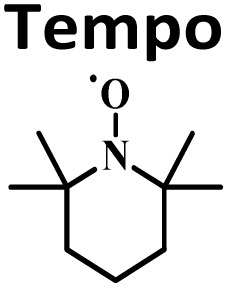	92%
2	Argon (Ar)	93%

a Conditions: 1a (0.3 mmol), 2 (0.15 mmol), H_5_IO_6_ (0.5 molar equivalent), H_2_O (0.5 mL), time (1 h), temperature (120 °C).

b Isolated yield.

To confirm the proposed mechanism, the reaction was performed under optimized conditions by replacing ethylene glycol (2a) with formaldehyde (5a), a known source of methylene carbon (Table 4, entry 1). The successful formation of the dimer proved that formaldehyde generated *in situ* from the Malaprade reaction reacts with 1a.

**Table 4:** Intermediary experiments^a^

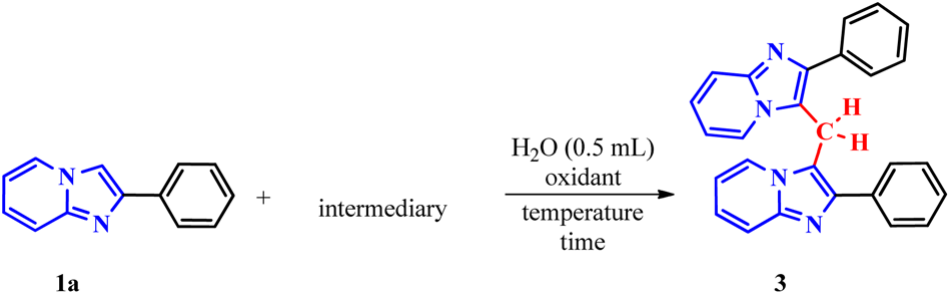			
Entry	Intermediary	Condition	3a, yield^b^ (%)
1	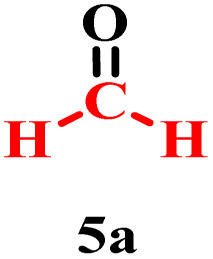	—	92%
2	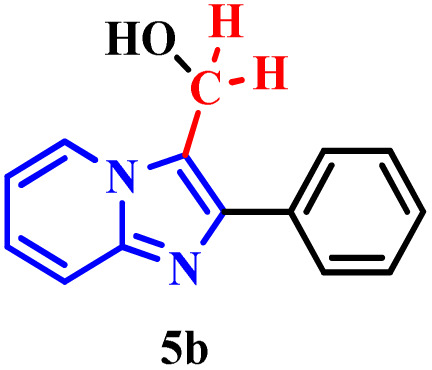	—	96%
3	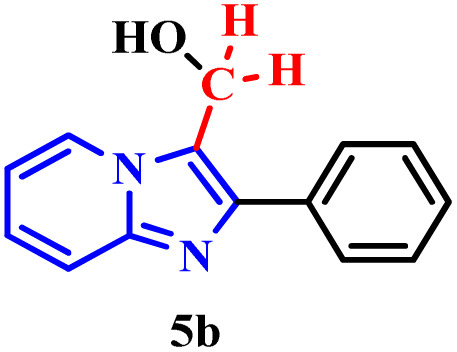	Only H_2_O 120 °C, 1 h	0%

a Conditions: 1a (0.3 mmol), 2 (molar equivalent), H_5_IO_6_ (0.5 molar equivalent), H_2_O (0.5 mL), time (1 h), temperature (120 °C).

b Isolated yield.

This hypothesis was further supported using (2-phenylimidazo[1,2-*a*]pyridin-3-yl)methanol (5b) under the same optimized conditions (Table 4, entry 2). The high yield obtained strongly suggests that 5b is a key reaction intermediate.

To elucidate the role of periodic acid, the reaction with alcohol 5b was repeated without the oxidant (Table 4, entry 3). No product was formed, confirming that an oxidizing agent is essential, even when starting from the proposed intermediate.

The importance of water as the solvent was also investigated. The reaction was conducted without water, using a three-fold excess of ethylene glycol (2a) to act as both reagent and solvent. Only trace amounts of product 3a were detected by TLC. The isolated quantity was insufficient for quantitative analysis or characterization by NMR.

Collectively, these results reinforce the reaction mechanism proposed by Franco.^17b^ It begins with the *in situ* formation of formaldehyde (5a) *via* oxidative cleavage of ethylene glycol (2a) according to the Malaprade reaction. This is followed by the formation of the hydroxylated intermediate (5b), which subsequently undergoes nucleophilic attack to form the dimer.

Based on all experimental evidence and previous reports, we propose a tentative mechanism for this transformation (Scheme 4). The mechanism begins with the Malaprade reaction, in which the 1,2-diol (2a) is cleaved by periodic acid to generate formaldehyde (5a) *in situ*, along with water and iodate. Formaldehyde (5a) then reacts with 1a to form the intermediate (2-phenylimidazo[1,2-*a*]pyridin-3-yl)methanol, denoted as III (5b). Subsequent dehydration of the protonated species (IV) yields the key electrophilic intermediate (V). Finally, nucleophilic attack by a second molecule of 2-phenylimidazo[1,2-*a*]pyridine (1a) on the methylene carbon of species V leads to the formation of the final product (3a).

**Scheme 4:**
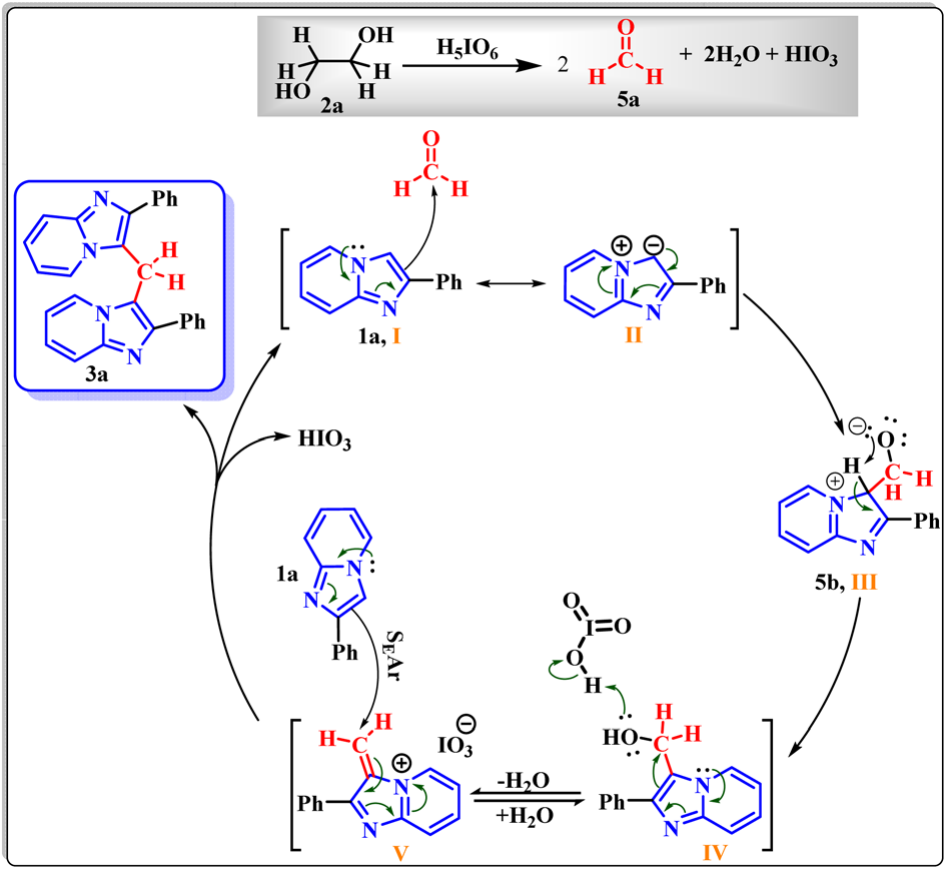
Mechanistic proposal.

## Conclusions

In this work, we developed a sustainable and efficient methodology for the methylenation of imidazoheteroarenes using ethylene glycol as a renewable and economical source of formaldehyde (C1), generated *in situ via* Malaprade oxidation. The protocol employs water as a non-toxic solvent, stoichiometric reagents, and mild reaction conditions, aligning with the principles of green chemistry. The method is highly regioselective, operationally simple, and delivers excellent yields (up to 97%) across a broad substrate scope, including imidazo[1,2-*a*]pyridines and imidazo[2,1-*b*]thiazoles. Mechanistic studies indicate an ionic pathway, with formaldehyde and a hydroxymethylated intermediate playing key role. Scalability was demonstrated (70% yield on gram-scale), highlighting the method's robustness for preparative applications.

This approach not only advances the synthetic toolbox for C–H functionalization of biologically relevant heterocycles but also underscores the potential of renewable feedstocks in sustainable chemistry. Future studies may unlock new pharmacological applications of these synthesized bis-heterocycles, leveraging the structural diversity enabled by this methodology.

## Author contributions

Conceptualization: M. S. F., S. S., J. R., and A. L. B.; methodology: M. S. F. and M. Y. G. W.; validation: M. S. F.; formal analysis: M. S. F., M. Y. G. W., I. M. O., and B. S. S.; investigation: M. S. F., M. Y. G. W., I. M. O., B. S. S., S. S., and J. R.; resources: S. S., J. R., and A. L. B.; data curation: M. S. F., M. Y. G. W., and I. M. O.; writing – original draft: M. S. F., and J. R.; writing – review and editing: S. S. and J. R.; visualization: S. S., J. R., and A. L. B.; supervision: S. S., J. R., and A. L. B.; project administration: S. S., J. R., and A. L. B.; funding acquisition: S. S., J. R., and A. L. B. All authors read and approved the final draft of the manuscript.

## Conflicts of interest

There are no conflicts to declare.

## Supplementary Material

RA-016-D5RA09947A-s001

## Data Availability

All experimental data supporting this article are available in the supplementary information (SI). Supplementary information: experimental procedures, substrates, general procedure for the preparation of compound 3 and 4, characterization data, and NMR spectra. See DOI: https://doi.org/10.1039/d5ra09947a.
