# Supplementary material for: Periodic acid-promoted methylenation of imidazoheteroarenes: a green approach using ethylene glycol as a C1 source
Source: RSC Adv. 2026 Mar 2;16(13):11937–43. doi: 10.1039/d5ra09947a (PMC12951599; doi:10.1039/d5ra09947a)
Supplement: RA-016-D5RA09947A-s001 [file RA-016-D5RA09947A-s001.pdf]

Supplementary Information (SI) for RSC Advances.
This journal is © The Royal Society of Chemistry 2026

Supporting Information

Periodic acid-Promoted Methylenation of Imidazoheteroarenes: A Green Approach Using Ethylene Glycol as C1 Source

Marcelo S. Franco,[a] Matheus Y. G. Watanabe,[a] Jhefferson S. Guilhermi,[b] Brunno S. Souza,[b] Sumbal Saba,[b]* Jamal Rafique,[b,c]* Antonio L. Braga[a]*

[a] Departamento de Química, Universidade Federal de Santa Catarina- UFSC, Florianópolis 88040-900, SC-Brazil.

[b] Laboratory of Sustainable Synthesis and Organochalcogen (LabSO), Instituto de Química, Universidade Federal de Goiás - UFG, Goiânia, 74690-900, GO-Brazil.

[c] Instituto de Química, Universidade Federal do Mato Grosso do Sul - UFMS, Campo Grande, 79074-460, MS-Brazil.

* Corresponding author: sumbal.saba@ufg.br (S.S.); braga.antonio@ufsc.br (A.L.B.); jamal.rafique@ufms.br (J.R.)

Table of Contents

I. EXPERIMENTAL PROCEDURES S2
I.I Thin layer chromatography S2
I.II Column chromatography S2
I.III Melting points S2
I.IV Nuclear magnetic resonance spectroscopy S2
I.V High Resolution Mass Spectrometry S2
II. SUBSTRATES S2
III. GENERAL PROCEDURE FOR THE PREPARATION OF COMPOUND **3**/**4**: . S3
IV. CHARACTERIZATION DATA S3
V. REFERENCES S9
VI. ^{1}H NMR AND ^{13}C NMR SPECTRA OF COMPOUNDS S10

I. EXPERIMENTAL PROCEDURES

Unless otherwise stated, all reagents and solvents were obtained from commercial sources and used without any further purification.

I.I Thin layer chromatography

Reaction monitoring and retention factor (*Rf*) determination were performed on pre-coated thin layer chromatography (TLC) sheets (ALUGRAM® Xtra SIL G/UV_{254}, MACHEREY-NAGEL, 0.20 mm thickness of layer) which were visualized either by quenching of ultraviolet or fluorescence light (λ_{max}= 254 and 366 nm, respectively) or by staining with iodine vapor and sprayed with vanillin–sulfuric acid solution, followed by heating the plate with a heat gun.[1]

I.II Column chromatography

Column chromatography was performed on silica gel (Silica gel 60, MACHEREY-NAGEL, 130 – 270 mesh particle size).

I.III Melting points

The melting points (MP) of the synthesized compounds were determined on a digital melting point instrument model MQAPF-301 (Microchemistry), using a heating rate of 2–3 °C min^{-1}. Data is expressed in degrees Celsius (°C).

I.IV Nuclear magnetic resonance spectroscopy

All proton nuclear magnetic resonance spectra (^{1}H NMR) and carbon-13 nuclear magnetic resonance spectra (^{13}C NMR) were obtained at 400 MHz or 100 MHz on a Bruker AVANCE DRX spectrometer. Spectra were recorded in $CDCl_3$. Chemical shifts (δ) were reported in part per million (ppm) in relation to tetramethylsilane (TMS, used as an internal standard for ^{1}H NMR spectra), and $CDCl_3$ (used as an internal standard for ^{13}C NMR spectra). For ^{1}H NMR spectra data are reported as follows: chemical shift (δ), multiplicity and coupling constant (*J*). Multiplicity of peaks are described as singlets (*s*), doublets (*d*), doublet of doublets (*dd*), doublet of doublets of doublets (*ddd*), triplets (*t*), doublet of triplets (*dt*), and multiplets (*m*).

I.V High Resolution Mass Spectrometry

HRMS analyzes were performed on a microTOF-QII mass spectrometer (Bruker), located at the Center for Structural Molecular Biology (CEBIME – UFSC). The spectrometer was operated in positive (+) and negative (-) ion modes, using Atmospheric Pressure Photoionization (APPI) or Electrospray Ionization (ESI) as the ionization mode. Data were processed in Bruker Data Analysis software version 4.0 and reported as m/z.

II. SUBSTRATES

The starting materials, imidazo[1,2-*a*]pyridines [2-8] and imidazo[2,1-*b*]thiazoles[9-10] were prepared according to the literature reports.

III. GENERAL PROCEDURE FOR THE PREPARATION OF COMPOUND 3/4:

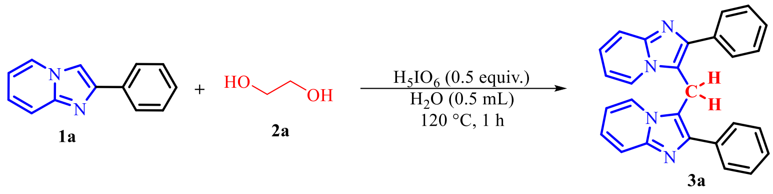

2-Phenylimidazo[1,2-*a*]pyridine **1a** or its derivatives (0.3 mmol), ethylene glycol (**2a**) (0.15 mmol), and water (0.5 mL) were added to a 20 mL Schlenk tube equipped with a magnetic stir bar. Periodic acid (0.15 mmol) was then added, and the tube was sealed. The reaction mixture was stirred for 1 hour at 120 °C in an oil bath. After the reaction time had elapsed, the system was allowed to cool to room temperature. The reaction mixture was transferred to a separation funnel, and saturated sodium bicarbonate ($NaHCO_3$) solution (10 mL) was added. The mixture was subsequently extracted with ethyl acetate (10 mL × 3). The organic phase was dried with anhydrous magnesium sulfate ($MgSO_4$), filtered, and concentrated under vacuum using a rotary evaporator. The crude product was purified by silica gel column chromatography with a suitable ethyl acetate/hexane mixture to provide the desired product.

IV. CHARACTERIZATION DATA

Bis(2-phenylimidazo[1,2-*a*]pyridin-3-yl)methane (3a)

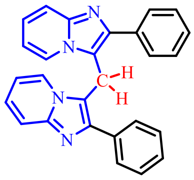

White solid, 57.1 mg, 95%; TLC (*Rf*): 0.23 (Ethyl Acetate, 100%); MP: 215-219 °C (216-218 °C)[14]; ^{1}H NMR (400 MHz, $CDCl_3$) δ (ppm): 7.80 – 7.76 (*m*, 4H), 7.54 – 7.48 (*m*, 6H), 7.45 – 7.40 (*m*, 2H), 7.33 (*dt*, J = 6.9, J = 1.1 Hz, 2H), 7.04 (*ddd*, J = 9.1, J = 6.8, J = 1.2 Hz, 2H), 6.46 (*td*, J = 6.8, J = 1.2 Hz, 2H), 4.98 (*s*, 2H); ^{13}C NMR (100 MHz, $CDCl_3$) δ (ppm): 145.0, 144.2, 134.4, 129.0, 128.9, 128.3, 124.4, 123.8, 117.5, 114.4, 112.3, 19.8.

Bis(6-methyl-2-phenylimidazo[1,2-*a*]pyridin-3-yl)methane (3b)

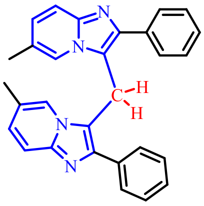

White solid, 62.6 mg, 94%; TLC (*Rf*): 0.36 (Hexane/Ethyl Acetate, 50:50); MP: 272-277 °C (273-275 °C)[14]; ^{1}H NMR (400 MHz, $CDCl_3$) δ (ppm): 7.84 (*d*, J = 7.9 Hz, 4H), 7.59 – 7.54 (*m*, 4H), 7.48 – 7.43 (*m*, 2H), 7.37 (*d*, J = 9.1 Hz, 2H), 7.02 (*s*, 2H), 6.83 (*dt*, J = 9.1, J = 1.6 Hz, 2H), 4.92 (*s*, 2H), 1.87 (*s*, 6H); ^{13}C NMR (100 MHz, $CDCl_3$) δ (ppm): 143.9, 143.5, 134.9, 129.0, 128.1, 127.4, 122.0, 121.6, 116.4, 114.4, 19.0, 17.9.

Bis(8-methyl-2-phenylimidazo[1,2-*a*]pyridin-3-yl)methane (3c)

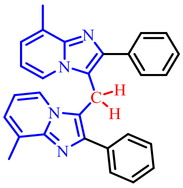

White solid, 107 mg, 76%; TLC (*Rf*): 0.26 (Hexane/Ethyl Acetate, 50:50); MP: 184-190 ºC (178-179ºC)[11]; ^{1}H NMR (400 MHz, $CDCl_3$) δ 7.76 – 7.73 (*m*, 4H), 7.49 – 7.42 (*m*, 4H), 7.39 – 7.34 (*m*, 2H), 7.20 (*d*, *J* = 6.8 Hz, 2H), 6.78 – 6.73 (*m*, 2H), 6.36 – 6.30 (*m*, 2H), 4.83 (*s*, 2H), 2.52 (*s*, 6H); ^{13}C NMR (100 MHz, $CDCl_3$) δ 145.1, 143.4, 134.4, 128.9, 128.5, 127.8, 127.1, 122.9, 121.4, 114.7, 112.1, 19.8, 16.9.

Bis(7-methyl-2-phenylimidazo[1,2-*a*]pyridin-3-yl)methane (3d)

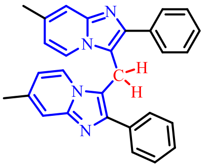

White solid, 60.6 mg, 97%; TLC (*Rf*): 0.32 (Hexane/Ethyl Acetate, 50:50); MP: 216-218 °C (215-217 ºC)[14]; ^{1}H NMR (400 MHz, $CDCl_3$) *δ* (ppm): 7.83 – 7.74 (*m*, 4H), 7.55 – 7.49 (*m*, 4H), 7.46 – 7.40 (*m*, 2H), 7.25 (*s*, 2H), 7.18 (*d*, *J* = 7.0 Hz, 2H), 6.27 (*dd*, *J* = 7.1, *J* = 1.6 Hz, 2H), 4.93 (*s*, 2H), 2.25 (*s*, 6H); ^{13}C NMR (100 MHz, $CDCl_3$) *δ* (ppm): 145.5, 143.7, 135.4, 134.6, 129.0, 128.9, 128.2, 123.1, 115.8, 115.0, 114.0, 21.2, 19.8.

Bis(2-(3,4-dimethoxyphenyl)imidazo[1,2-*a*]pyridin-3-yl)methane (3e)

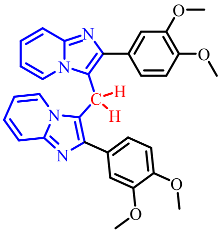

White solid, 65.6 mg, 84%; TLC (*Rf*): 0.05 (Hexane/Ethyl acetate, 50:50); MP: 150-155 °C (192-193 ºC)[12]; ^{1}H NMR (200 MHz, $CDCl_3$) *δ* (ppm): 7.55 (*d*, *J* = 9.0 Hz, 2H), 7.43 – 7.36 (*m*, 4H), 7.28 (*dd*, *J* = 8.1, *J* = 1.7 Hz, 2H), 7.12 – 7.02 (*m*, 2H), 6.99 (*d*, *J* = 8.3 Hz, 2H), 6.49 (*t*, *J* = 6.7 Hz, 2H), 4.98 (*s*, 2H), 3.98 (*s*, 6H), 3.96 (*s,* 6H); ^{13}C NMR (50 MHz, $CDCl_3$) *δ* (ppm): 149.5, 149.3, 144.8, 143.9, 127.0, 124.5, 123.9, 121.3, 117.2, 114.1, 112.4, 112.2, 111.2, 56.2, 56.1, 19.8; HRMS (ESI+) m/z:$[M+H]^+$ calc. for $C_{31}H_{29}N_4O_4$, 521.2183; found: 521.2195.

Bis(2-(3-methoxyphenyl)imidazo[1,2-*a*]pyridin-3-yl)methane (3f)

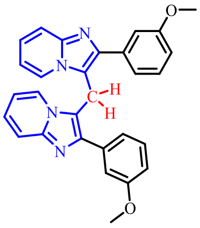

Beige solid, 60.1 mg, 87%; TLC (*Rf*): 0.16 (Hexane/Ethyl Acetate, 50:50); MP: 120-134 °C; ^{1}H NMR (200 MHz, $CDCl_3$) *δ* (ppm): 7.56 – 7.46 (*m*, 2H), 7.44 – 7.27 (*m*, 8H), 7.14 – 6.88 (*m*, 4H),

6.60 – 6.31 (*m*, 2H), 4.97 (*s*, 2H), 3.86 (*s*, 6H); ^{13}C NMR (50 MHz, $CDCl_3$) *δ* (ppm): 160.0, 144.9, 144.0, 135.7, 129.8, 124.4, 123.9, 121.4, 117.4, 114.5, 114.3, 114.2, 112.3, 55.4, 19.7; HRMS (ESI+) m/z: $[M+H]^+$ calc. for $C_{29}H_{25}N_4O_2$, 461.1972; found: 461.1979.

Bis(2-(4-fluorophenyl)imidazo[1,2-*a*]pyridin-3-yl)methane (3g)

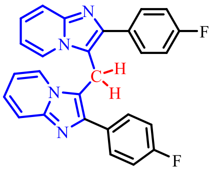

White solid, 60.4 mg, 92%; TLC (*Rf*): 0.23 (Hexane/Ethyl Acetate, 50:50); MP: 189-192 °C (190-192 ºC)[14]; ^{1}H NMR (400 MHz, $CDCl_3$) *δ*: 7.72 – 7.63 (*m*, 4H), 7.53 (*dt*, *J* = 9.1, 1.0 Hz, 2H), 7.37 (*dt*, *J* = 6.9, 1.0 Hz, 2H), 7.18 – 7.11 (*m*, 4H), 7.09 (*ddd*, *J* = 9.0, 6.8, 1.2 Hz, 2H), 6.54 (*td*, *J* = 6.8, 1.2 Hz, 2H), 4.87 (*s*, 2H); ^{13}C NMR (100 MHz, $CDCl_3$) *δ*: 162.7 (*d*, $J_{F\text{-}C}$ = 248.1 Hz), 144.9, 143.3, 130.5 (*d*, $J_{F\text{-}C}$ = 8.2 Hz), 130.3 (*d*, $J_{F\text{-}C}$ = 3.3 Hz), 124.5, 123.5, 117.5, 115.7 (*d*, $J_{F\text{-}C}$ = 21.6 Hz), 113.9, 112.5, 19.9.

Bis(2-(4-chlorophenyl)imidazo[1,2-*a*]pyridin-3-yl)methane (3h)

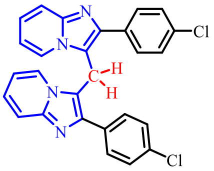

White solid, 57.8 mg, 82%; TLC (*Rf*): 0.34 (Hexane/Ethyl Acetate, 50:50); MP: 250-255 °C (250-252 ºC)[11]; ^{1}H NMR (200 MHz, $CDCl_3$) *δ* (ppm): 7.63 (*d*, ^3J = 8.4 Hz, 4H), 7.53 (*d*, ^3J = 9.1 Hz, 2H), 7.46 – 7.32 (*m*, 6H), 7.09 (*ddd*, *J* = 9.0, *J* = 6.8, *J* = 0.9 Hz, 2H), 6.55 (*td*, *J* = 6.8, *J* = 1.1 Hz, 2H), 4.88 (*s*, 2H); ^{13}C NMR (50 MHz, $CDCl_3$) *δ* (ppm): 145.1, 143.2, 134.3, 132.7, 130.0, 129.0, 124.7, 123.5, 117.7, 114.1, 112.7, 20.1.

Bis(6-chloro-2-phenylimidazo[1,2-*a*]pyridin-3-yl)methane (3i)

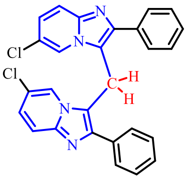

White solid, 37.3 mg, 53%; TLC (*Rf*): 0.64 (Hexane/Ethyl Acetate, 50:50); MP: 225-230 °C (234-236 ºC)[11]; ^{1}H NMR (400 MHz, $CDCl_3$) *δ* (ppm): 7.77 – 7.72 (*m*, 4H), 7.59 – 7.54 (*m*, 4H), 7.51 – 7.47 (*m*, 2H), 7.45 – 7.42 (*m*, 2H), 7.34 – 7.23 (*m*, 2H), 7.01 (*dd*, ^3J = 9.5, ^4J = 1.9 Hz, 2H), 4.90 (*s*, 2H); ^{13}C NMR (100 MHz, $CDCl_3$) *δ* (ppm): 145.4, 143.5, 133.7, 129.3, 129.1, 128.8, 126.0, 122.0, 120.7, 117.8, 114.8, 19.2.

Bis(2-(4-bromophenyl)imidazo[1,2-*a*]pyridin-3-yl)methane (3j)

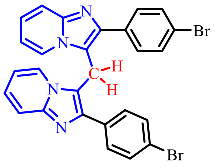

White solid, 63.6 mg, 76%; TLC (*Rf*): 0.64 (Hexane/Ethyl Acetate, 50:50); MP: 255-260 °C (254-255 ºC)[11]; ^{1}H NMR (400 MHz, $CDCl_3$) *δ* (ppm): 7.56 (*s*, 8H), 7.54 (*d*, *J* = 9.1 Hz, 2H), 7.36 (*d*, *J*

= 6.9 Hz, 2H), 7.10 (*ddd*, *J* = 8.8, *J* = 6.8, *J* = 0.9 Hz, 2H), 6.56 (*td*, *J* = 6.8, *J* = 0.8 Hz, 2H), 4.87 (*s*, 2H); ^{13}C NMR (100 MHz, $CDCl_3$) δ (ppm): 145.1, 143.2, 133.1, 131.9, 130.2, 124.7, 123.5, 122.6, 117.7, 114.1, 112.8, 20.1.

Bis(2-(5-chlorothiophen-2-yl)imidazo[1,2-*a*]pyridin-3-yl)methane (3k)

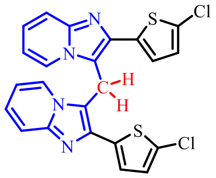

Pale brown solid, 53 mg, 73%; TLC (*Rf*): 0.60 (Hexane/Ethyl Acetate, 50:50); MP: 247-250 °C (253-254 ºC)[13]; ^{1}H NMR (400 MHz, $CDCl_3$) δ (ppm): 7.53 (d, *J* = 9.0 Hz, 2H), 7.50 (s, 2H), 7.28 (d, *J* = 3.9 Hz, 2H), 7.13 – 7.08 (m, 2H), 6.99 (d, *J* = 3.9 Hz, 2H), 6.58 (t, *J* = 6.8 Hz, 2H), 5.01 (s, 2H); ^{13}C NMR (101 MHz, $CDCl_3$) δ (ppm): 145.4, 137.8, 135.8, 131.3, 127.2, 125.3, 125.0, 123.7, 117.6, 113.4, 113.3, 20.4; HRMS (ESI+) m/z:[M+H]$^{+}$ calc. for $C_{23}H_{15}Cl_2N_4S_2$, 481.0110; found: 481.0115.

4,4'-(Methylenebis(imidazo[1,2-*a*]pyridine-3,2-diyl))dibenzonitrile (3l)

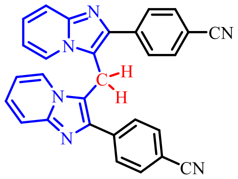

White solid, 57.8 mg, 86%; TLC (*Rf*): 0.31 (Hexane/Ethyl Acetate, 50:50); MP: 294-296 °C (295-297 ºC)[14]; ^{1}H NMR (200 MHz, $CDCl_3$) δ (ppm): 7.63 (*d*, *J* = 8.5 Hz, 4H), 7.53 (*d*, *J* = 9.1 Hz, 2H), 7.44 – 7.33 (*m*, 6H), 7.09 (*ddd*, *J* = 8.9, *J* = 6.8, *J* = 1.0 Hz, 2H), 6.55 (*td*, *J* = 6.8, *J* = 0.9 Hz, 2H), 4.87 (*s*, 2H); ^{13}C NMR (50 MHz, $CDCl_3$) δ (ppm): 145.1, 143.2, 134.3, 132.7, 130.0, 129.0, 124.7, 123.5, 117.7, 114.1, 112.7, 20.1.

Bis(2-(4-(methylsulfonyl)phenyl)imidazo[1,2-*a*]pyridin-3-yl)methane (3m)

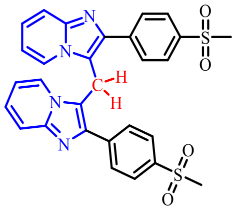

Pale red-brown solid, 56.3 mg, 67%; TLC (*Rf*): 0.083 (Hexane/Ethyl Acetate, 50:50); MP: 273-275 °C (274-275 °C)[11]; ^{1}H NMR (400 MHz, $CDCl_3$) δ (ppm): 7.93 (d, *J* = 8.4 Hz, 4H), 7.79 (d, *J* = 8.6 Hz, 4H), 7.57 (d, *J* = 9.1 Hz, 2H), 7.50 (d, *J* = 6.9 Hz, 2H), 7.20 (d, *J* = 6.8 Hz, 2H), 6.71 (td, *J* = 6.8, 1.2 Hz, 2H), 4.93 (s, 2H), 3.09 (s, 6H); ^{13}C NMR (101 MHz, $CDCl_3$) δ (ppm): 145.0, 142.3, 139.6, 139.2, 129.0, 127.3, 125.1, 123.0, 117.8, 114.4, 113.1, 44.4, 29.5.

Bis(2-(4-methoxyphenyl)-7-methylimidazo[1,2-*a*]pyridin-3-yl)methane (3n)

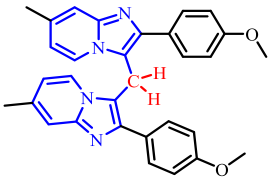

White solid, 63.3 mg, 87%; TLC (*Rf*): 0.60 (Hexane/Ethyl Acetate, 50:50); MP: 203-205 ºC; ^{1}H NMR (400 MHz, $CDCl_3$) δ (ppm): 7.74 – 7.70 (*m*, 4H), 7.23 (*s*, 2H), 7.19 (*d*, *J* = 7.1 Hz, 2H), 7.06 – 7.02 (*m*, 4H), 6.26 (*dd*, *J* = 7.1, *J* = 1.5 Hz, 2H), 4.87 (*s*, 2H), 3.86 (*s*, 6H), 2.24 (*s*, 6H); ^{13}C NMR (100 MHz, $CDCl_3$) δ (ppm): 159.5, 145.2, 143.4, 135.0, 130.0, 127.0, 123.0, 115.5, 114.6, 114.2, 113.4, 55.3, 21.1, 19.7; HRMS (ESI+) m/z: $[M+H]^+$ calc. for $C_{31}H_{29}N_4O_2$, 489.2285; found: 489.2291.

Bis(imidazo[1,2-a]pyridin-3-yl)methane (3o)

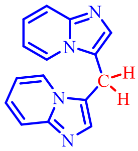

White solid, 36 mg, 36%; TLC (*Rf*): 0.30 (Hexane/Ethyl Acetate, 50:50); MP: 235-239 °C (219-221 ºC)[11]; ^{1}H NMR (400 MHz, $CDCl_3$) δ (ppm): δ 7.93 (d, *J* = 6.9 Hz, 2H), 7.64 (d, *J* = 9.2 Hz, 2H), 7.49 (s, 2H), 7.24 – 7.15 (m, 2H), 6.80 (t, J = 6.9 Hz, 2H), 4.52 (s, 2H); ^{13}C NMR (101 MHz, $CDCl_3$) δ (ppm): 146.3, 132.7, 124.2, 123.3, 118.2, 112.7, 20.2.

Bis(2-methyl-6-phenylimidazo[2,1-*b*]thiazol-5-yl)methane (4a)

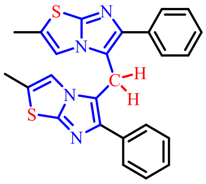

White solid, 53.9 mg, 84%; TLC (*Rf*): 0.53 (Hexane/Ethyl acetate, 50:50); MP: 235-239 °C; ^{1}H NMR (400 MHz, $CDCl_3$) δ (ppm): 7.74 – 7.71 (m, 4H), 7.53 – 7.48 (m, 4H), 7.42 – 7.37 (m, 2H), 6.19 – 6.17 (m, 2H), 4.72 (s, 2H), 2.17 – 2.16 (m, 6H); ^{13}C NMR (100 MHz, $CDCl_3$) δ (ppm): 148.9, 143.3, 134.8, 129.0, 128.1, 127.7, 126.6, 117.4, 114.1, 21.2, 14.2; HRMS (ESI+) m/z:$[M+H]^+$ calc. for $C_{25}H_{21}N_4S_2$, 441.1202; found: 441.1208.

Bis(3-methyl-6-phenylimidazo[2,1-*b*]thiazol-5-yl)methane (4b)

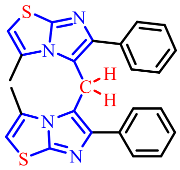

White solid, 56.5 mg, 85%; TLC (*Rf*): 0.35 (Hexane/Ethyl acetate, 50:50); MP: 198-200 °C; ^{1}H NMR (400 MHz, $CDCl_3$) δ (ppm): 7.27 – 7.23 (m, 4H), 7.20 – 7.15 (m, 6H), 6.18 (d, J = 1.2 Hz, 2H), 4.80 (s, 2H), 2.33 – 2.33 (s, 6H); ^{13}C NMR (100 MHz, $CDCl_3$) δ (ppm): 149.6, 145.6, 134.0, 129.0, 127.9, 127.6, 127.1, 119.0, 107.4, 22.0, 14.4; HRMS (ESI+) m/z:$[M+H]^+$ calc. for $C_{25}H_{21}N_4S_2$, 441.1202; found: 441.1208.

Bis(6-(4-methoxyphenyl)-2-methylimidazo[2,1-*b*]thiazol-5-yl)methane (4c)

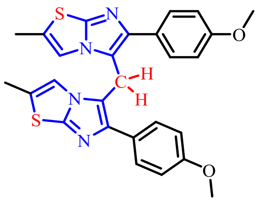

Pale brown solid, 67 mg, 89%; TLC (*Rf*): 0.65 (Hexane/Ethyl acetate, 50:50); MP: 210-215 °C; ^{1}H NMR (400 MHz, $CDCl_3$) δ (ppm): 7.66 – 7.62 (m, 4H), 7.07 – 6.99 (***m*****,** 4H), 6.24 – 6.22 (m, 2H), 4.64 (*s*, 2H), 3.87 (*s*, 6H), 2.18 – 2.17 (m, 6H); ^{13}C NMR (100 MHz, $CDCl_3$) δ (ppm): 159.2, 148.6, 143.0, 129.3, 127.3, 126.3, 116.7, 114.4, 114.1, 55.5, 21.2, 14.2; HRMS (ESI+) m/z:[M+H]$^{+}$ calc. for $C_{27}H_{25}N_4O_2S_2$, 501.1413; found: 501.1419.

Bis(6-(3-bromophenyl)-2-methylimidazo[2,1-*b*]thiazol-5-yl)methane (4d)

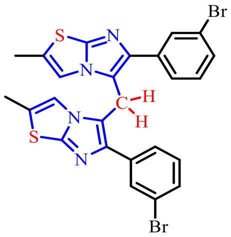

White solid, 52 mg, 58%; TLC (*Rf*): 0.61 (Hexane/Ethyl acetate, 50:50); MP: 180-185 °C; ^{1}H NMR (400 MHz, $CDCl_3$) δ (ppm): 7.88 (*t*, *J* = 1.7 Hz, 2H), 7.58 (*ddd*, *J* = 7.7, *J* = 1.5, *J* = 1.1 Hz, 2H), 7.50 (*ddd*, *J* = 8.0, *J* = 2.0, *J* = 1.0 Hz, 2H), 7.33 (*t*, *J* = 7.9 Hz, 2H), 6.23 – 6.22 (*m*, 2H), 4.65 (*s*, 2H), 2.21 – 2.21 (m, 6H); ^{13}C NMR (100 MHz, $CDCl_3$) δ (ppm): 149.1, 141.9, 136.6, 131.0, 130.6, 130.3, 127.4, 126.3, 123.1, 117.3, 113.7, 21.2, 14.2; HRMS (ESI+) m/z:[M+H]$^{+}$ calc. $C_{25}H_{19}Br_2N_4S_2$, 598.9392; found: 598.9398.

4,4'-(5,5'-methylenebis(2-methylimidazo[2,1-b]thiazole-6,5-diyl))dibenzonitrile (4e)

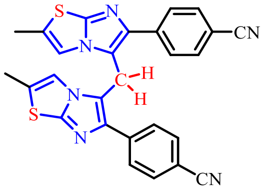

White solid, 46.4 mg, 63%; TLC (*Rf*): 0.36 (Hexane/Ethyl acetate, 50:50); MP: 200-206 °C; ^{1}H NMR (400 MHz, $CDCl_3$) δ (ppm): 7.81 (m, 4H), 7.74 (m, 4H), 6.33 (d, *J* = 1.5 Hz, 2H), 4.74 (s, 2H), 2.26 – 2.26 (m, 6H); ^{13}C NMR (101 MHz, $CDCl_3$) δ (ppm): 149.7, 141.8, 138.9, 132.7, 128.5, 128.1, 118.9, 117.6, 113.3, 111.1, 22.1, 14.3; HRMS (ESI+) m/z:[M+H]$^{+}$ calc. for $C_{27}H_{19}N_6S_2$, 492.1141 found: 492.1140.

V. REFERENCES

1. H. Jork, W. Funk, W. Fischer, H. Wimmer, *Thin-Layer Chromatography Reagents Detect. Methods,* Vol.1a, VHC, Weinheim, **1990**, p. 497.
2. H. Huang, X. Ji, X. Tang, M. Zhang, X. Li, H. Jiang, *Org. Lett.* **2013**, *15*, 6254–6257.
3. F.-J. Wang, H. Xu, M. Xin, Z. Zhang, *Mol. Divers.* **2016**, *20*, 659–666.
4. S. K. Samanta, M. K. Bera, *Org. Biomol. Chem.* **2019**, *17*, 6441–6449.
5. J. L. Bescont, C. B.-Patient, S. Piguel, *Eur. J. Org. Chem.* **2020**, 2101–2109.
6. N. Gunaganti, A. Kharbanda, N. R. Lakkaniga, L. Zhang, R. Cooper, H.-y. Li, B. Frett, *Chem. Commun.* **2018**, *54*, 12954–12957.
7. Q. Li, M. Zhou, L. Han, Q. Cao, X. Wang, L. Zhao, J. Zhou, H. Zhang, *Chem. Biol. Drug. Des.* **2015**, *86*, 849–856.
8. A. J. Stasyuk, M. Banasiewicz, M. K. Cyranski, D. T. Gryko, *J. Org. Chem.* **2012**, *77*, 5552–5558
9. T. Pyl, R. Giebelmann, H. Beyer, *Liebigs Ann. Chem.* **1961**, *643*, 145–153.
10. A. Kamal, D. Dastagiri, M. J. Ramaiah, J. S. Reddy, E. V. Bharathi, C. Srinivas, S. N. C. V. L. Pushpavalli, D. Pal, M. P.-Bhadra, *Chem. Med. Chem.* **2010**, *5*, 1937–1947.
11. P. Liu, Z. Shen, Y. Yuan, P. Sun. *Org. Biomol. Chem.* **2016**, *14* (27), 6523–6530.
12. P. Kaswan, N. K. Nandwana, B. DeBoef, A. Kumar. *Advanced Synthesis & Catalysis* **2016**, *358* (13), 2108–2115.
13. M. S. Franco, S. Saba, J. Rafique, A. L. Braga. *Angewandte Chemie International Edition* **2021**, *60* (34), 18454–18460.
14. P. P. S. Patel, D. Anand, R. K. Maurya, P. P. Yadav. *J. Org. Chem.* **2016**, *81* (17), 7626–7634.

VI. ^{1}H NMR AND ^{13}C NMR SPECTRA OF COMPOUNDS

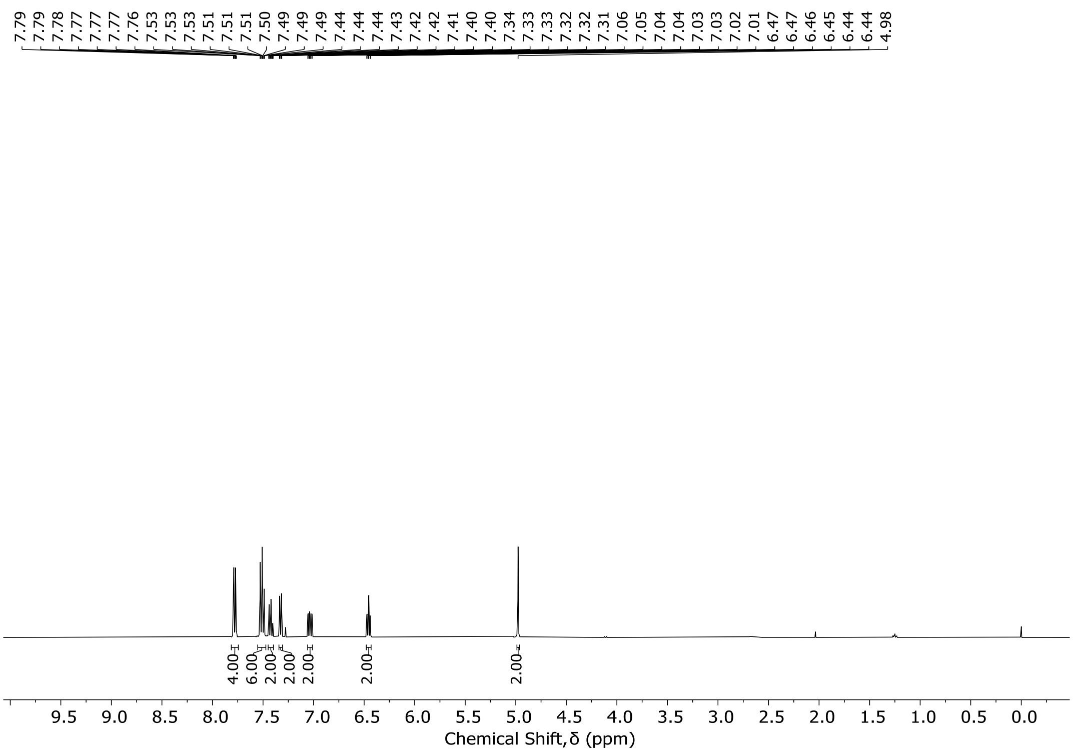

^{1}H NMR Spectra of **3a** in $CDCl_3$ at 400 MHz

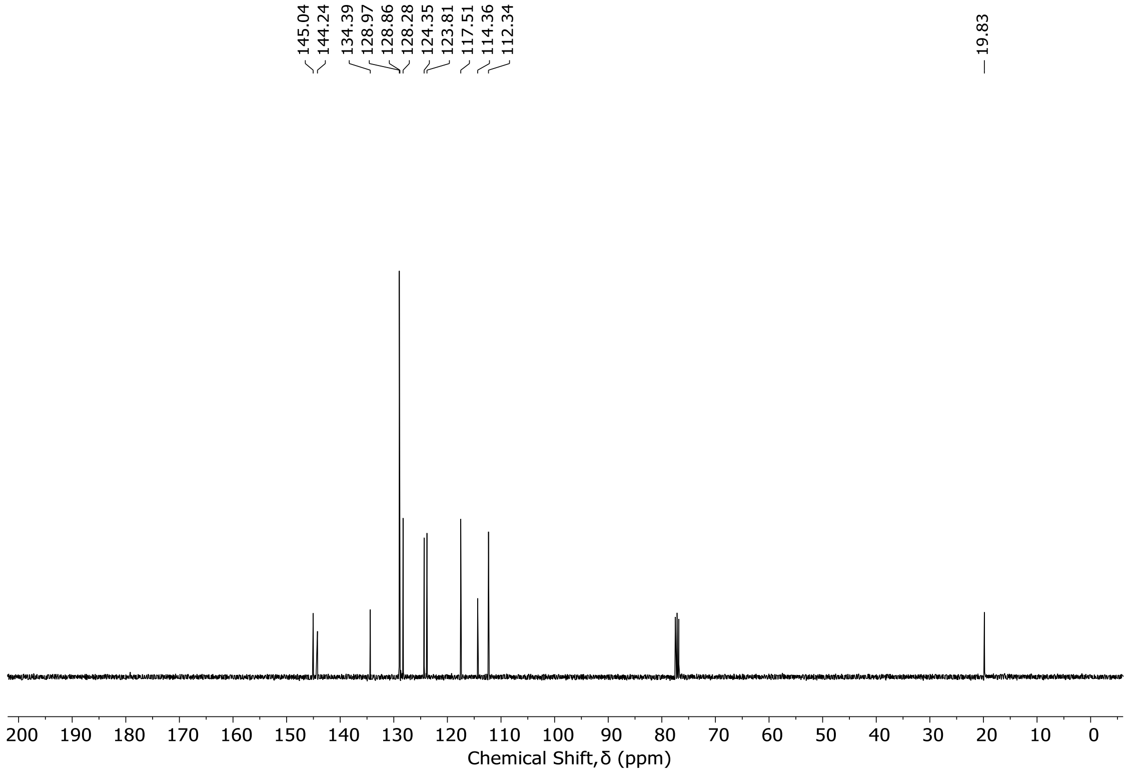

^{13}C NMR Spectra of **3a** in $CDCl_3$ at 100 MHz

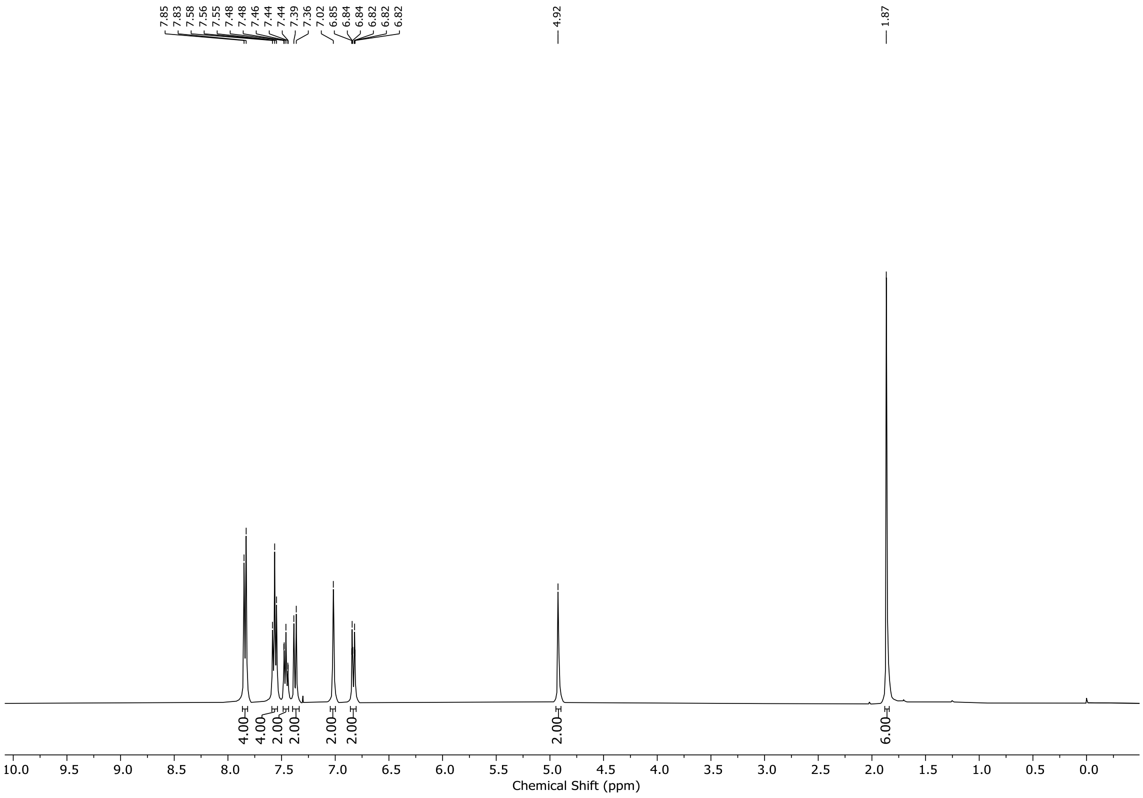

^{1}H NMR Spectra of **3b** in $CDCl_3$ at 400 MHz

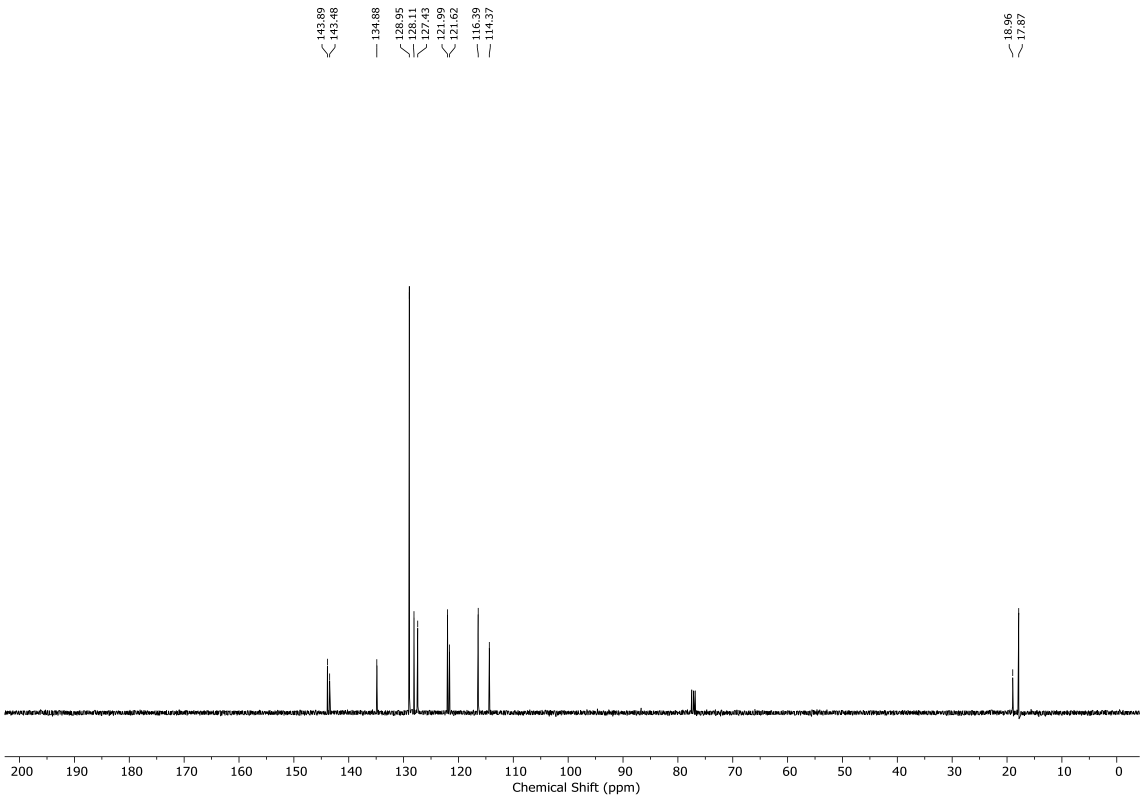

^{13}C NMR Spectra of **3b** in $CDCl_3$ at 100 MHz

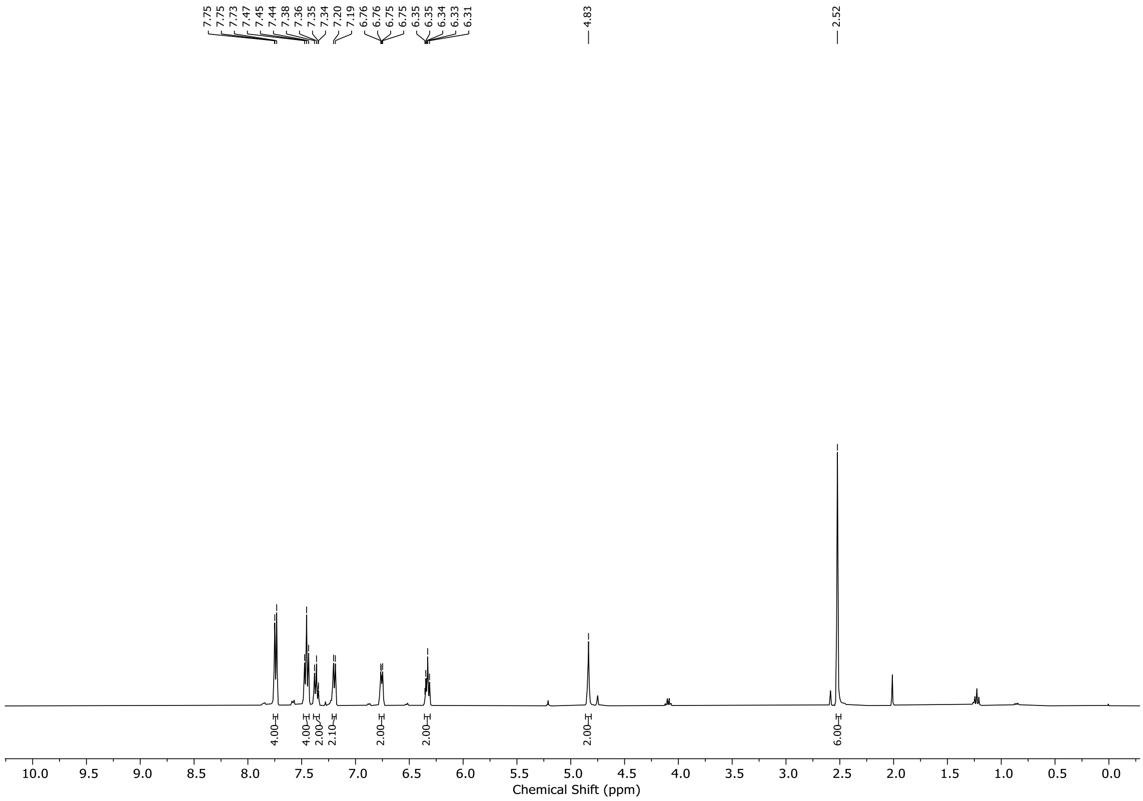

^{1}H NMR Spectra of **3c** in $CDCl_3$ at 400 MHz

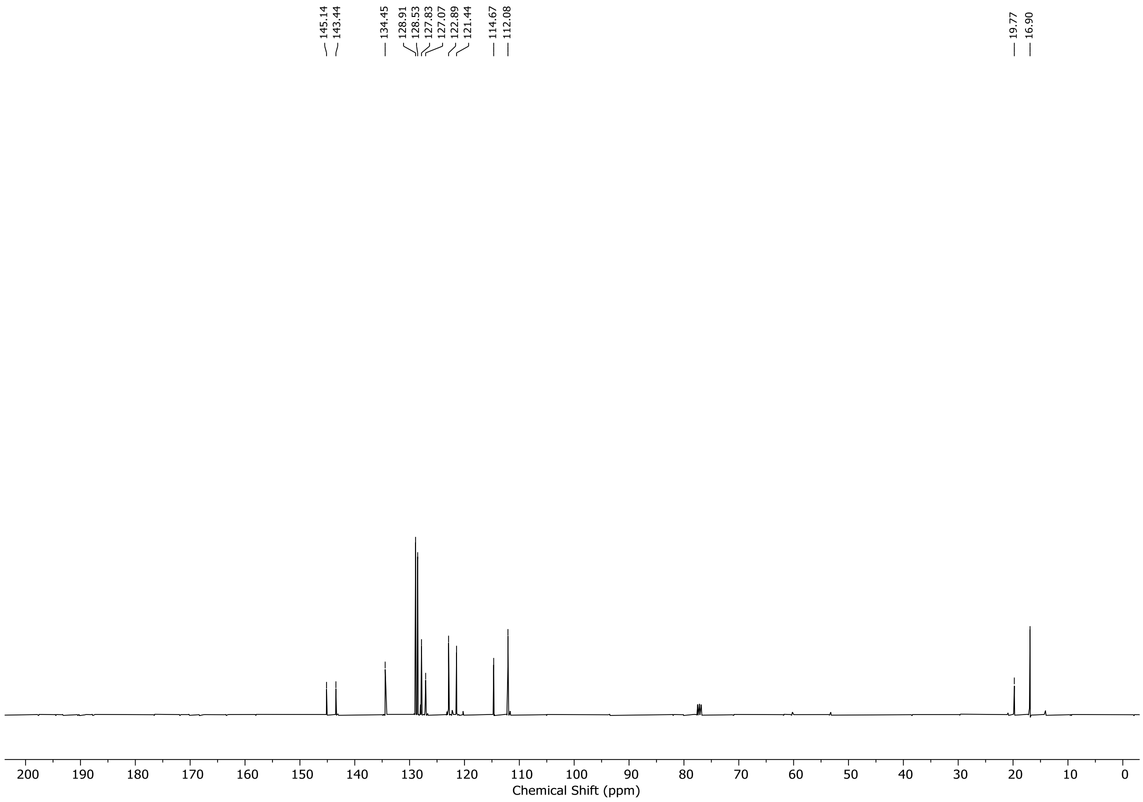

^{13}C NMR Spectra of **3c** in $CDCl_3$ at 100 MHz

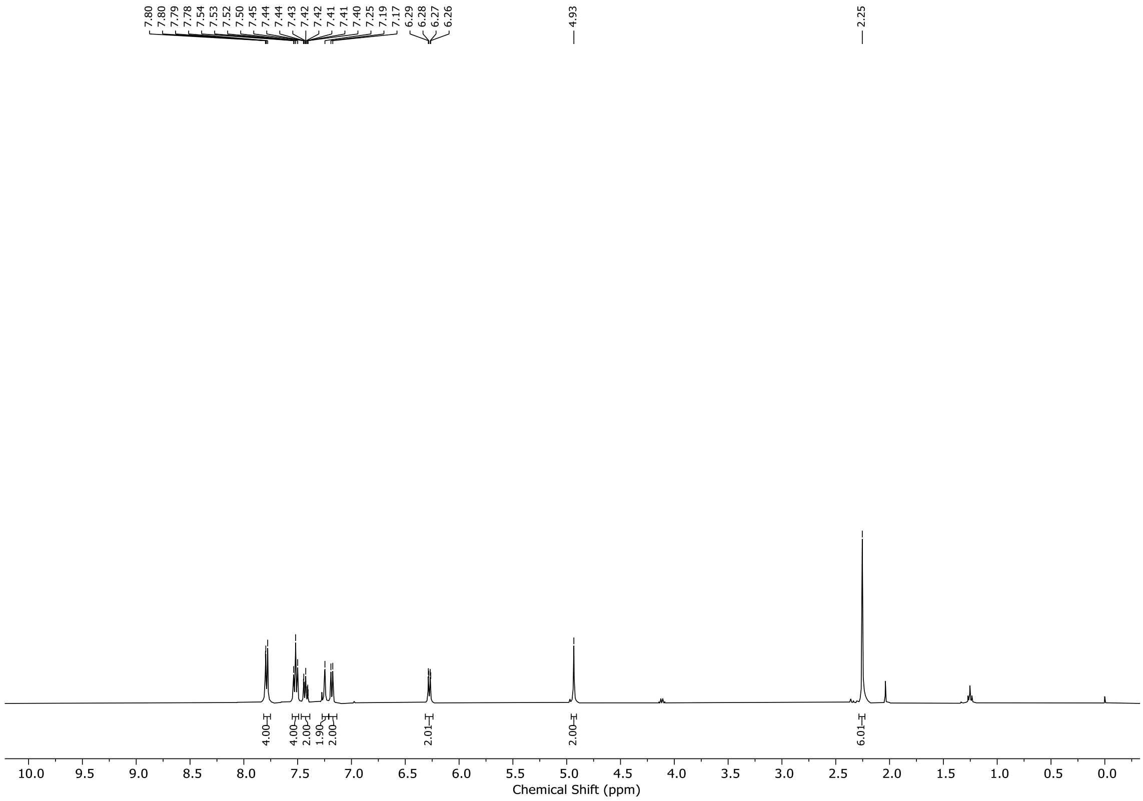

^1H NMR Spectra of **3d** in $CDCl_3$ at 400 MHz

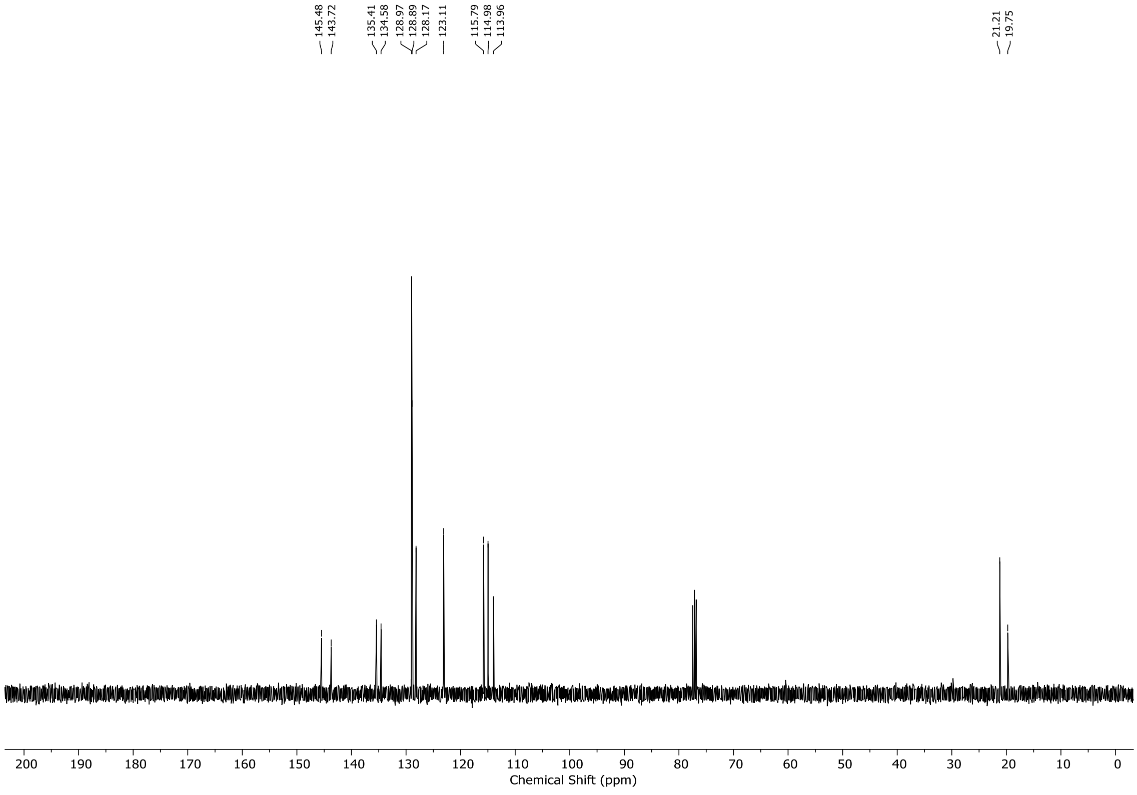

^{13}C NMR Spectra of **3d** in $CDCl_3$ at 100 MHz

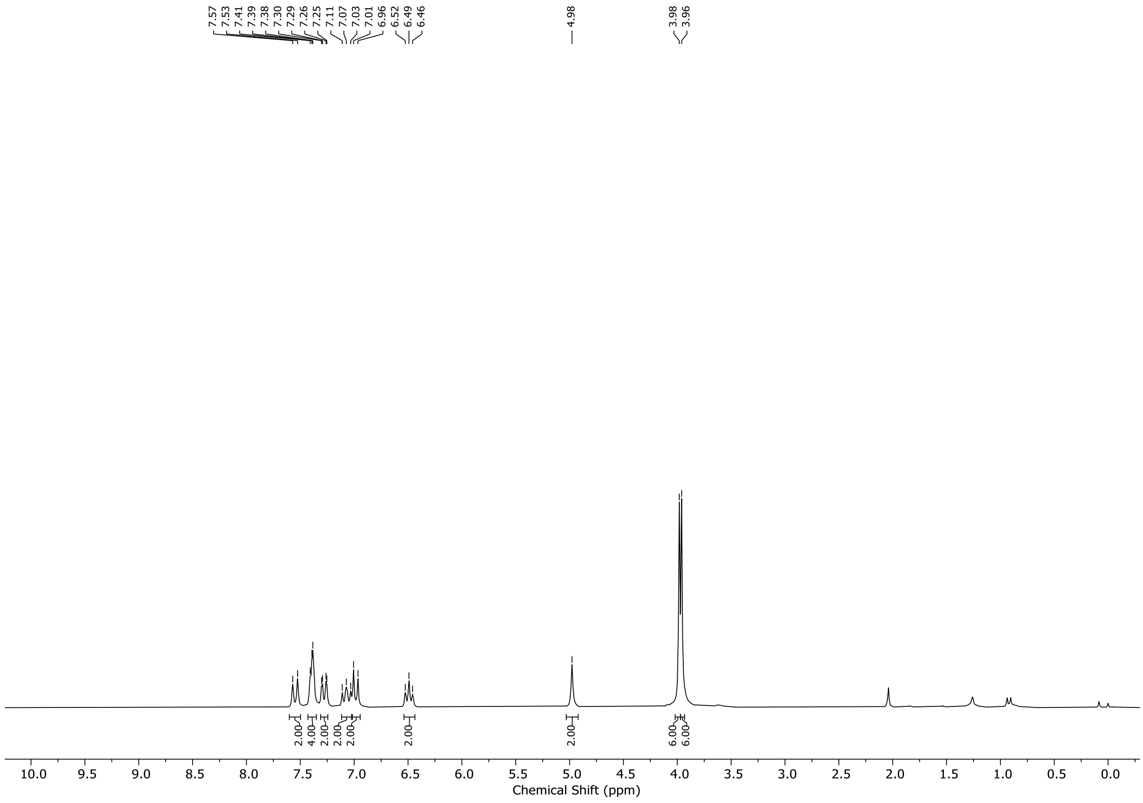

^{1}H NMR Spectra of **3e** in $CDCl_3$ at 200 MHz

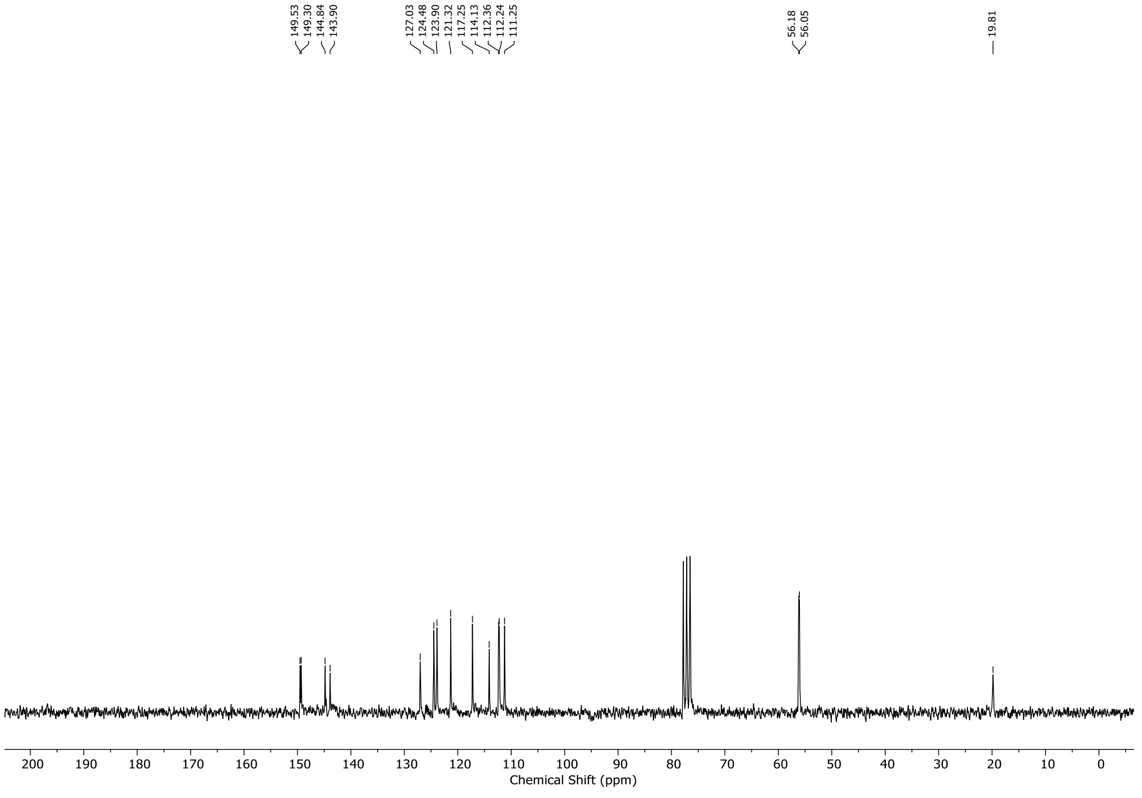

^{13}C NMR Spectra of **3e** in $CDCl_3$ at 50 MHz

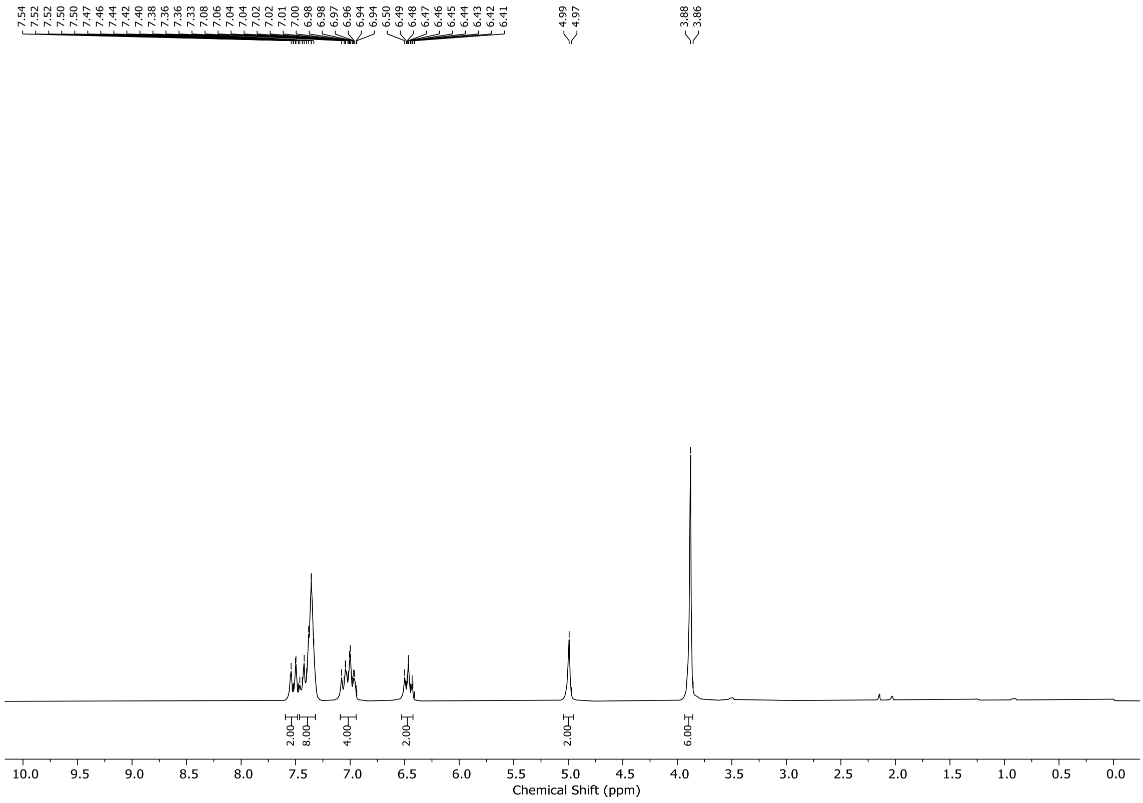

^{1}H NMR Spectra of **3f** in $CDCl_3$ at 200 MHz

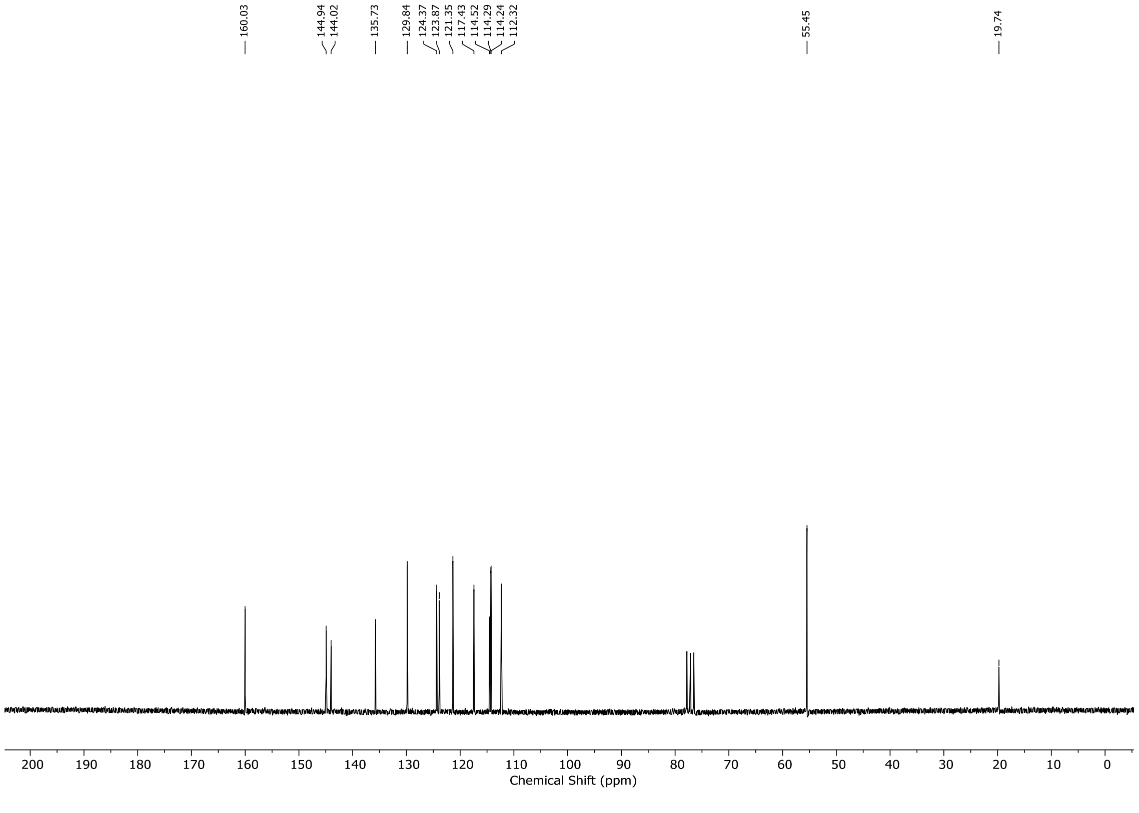

^{13}C NMR Spectra of **3f** in $CDCl_3$ at 50 MHz

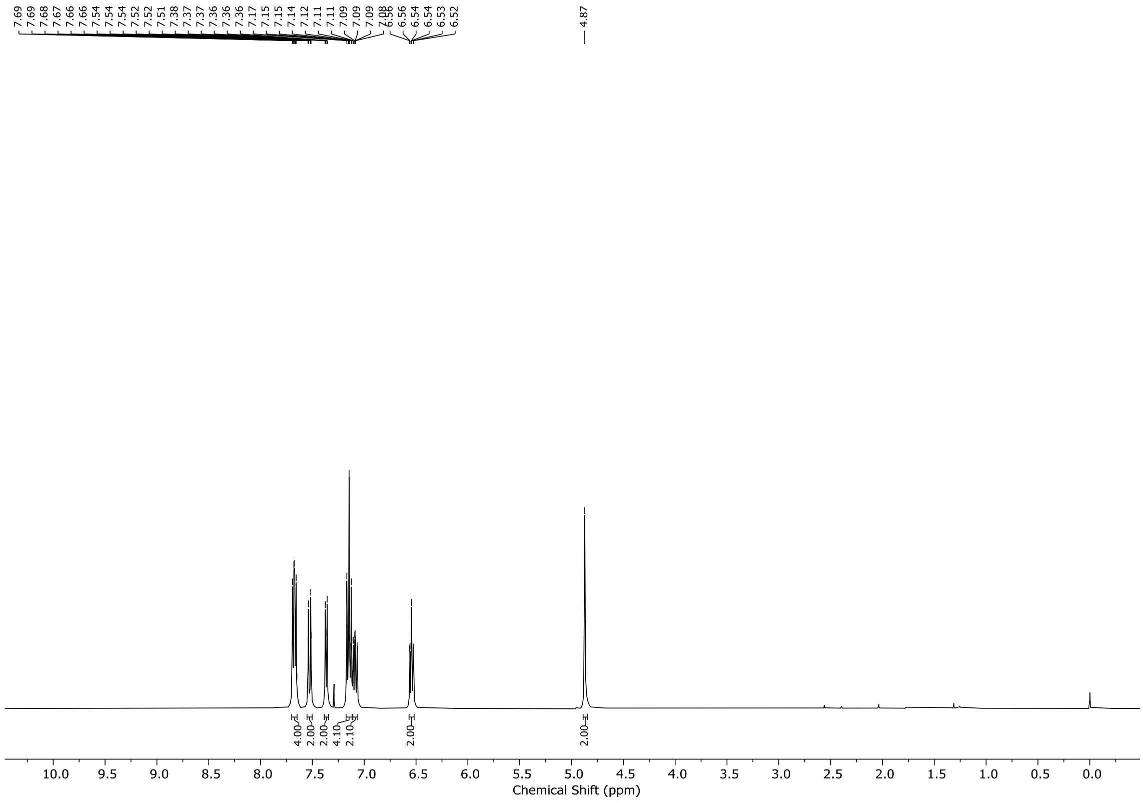

^{1}H NMR Spectra of **3g** in $CDCl_3$ at 400 MHz

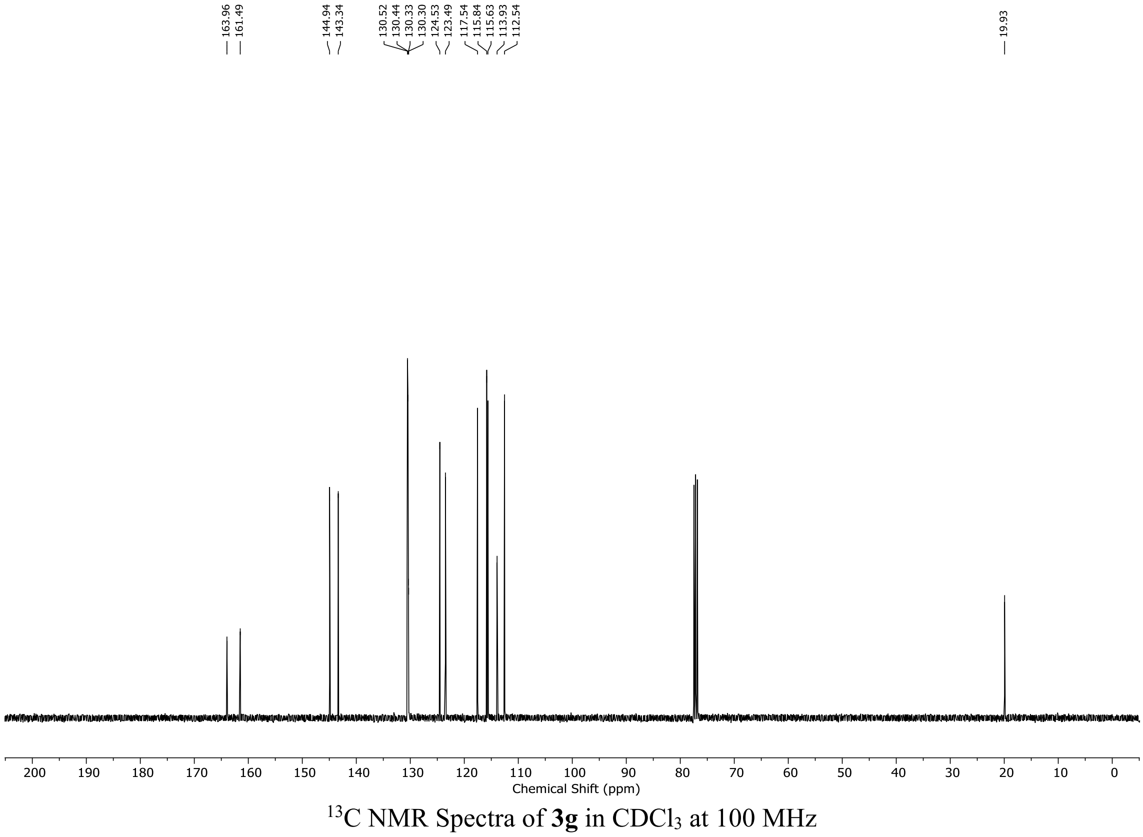

^{13}C NMR Spectra of **3g** in $CDCl_3$ at 100 MHz

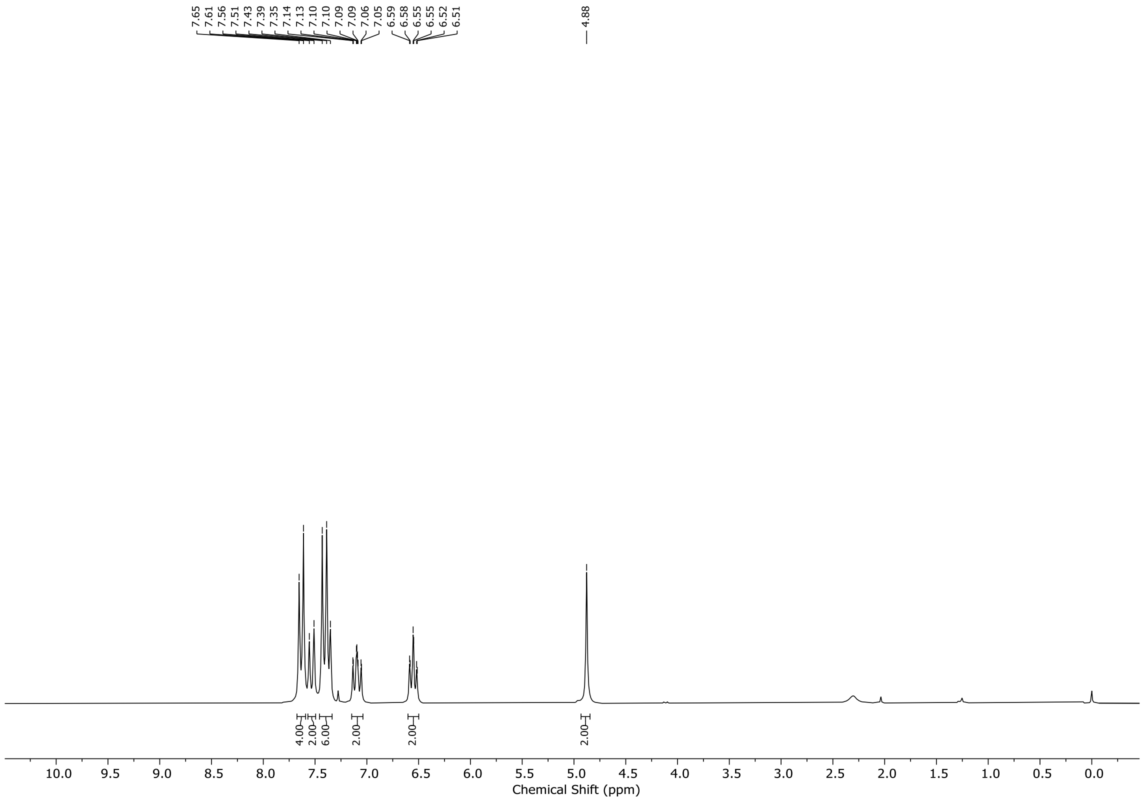

^{1}H NMR Spectra of **3h** in $CDCl_3$ at 200 MHz

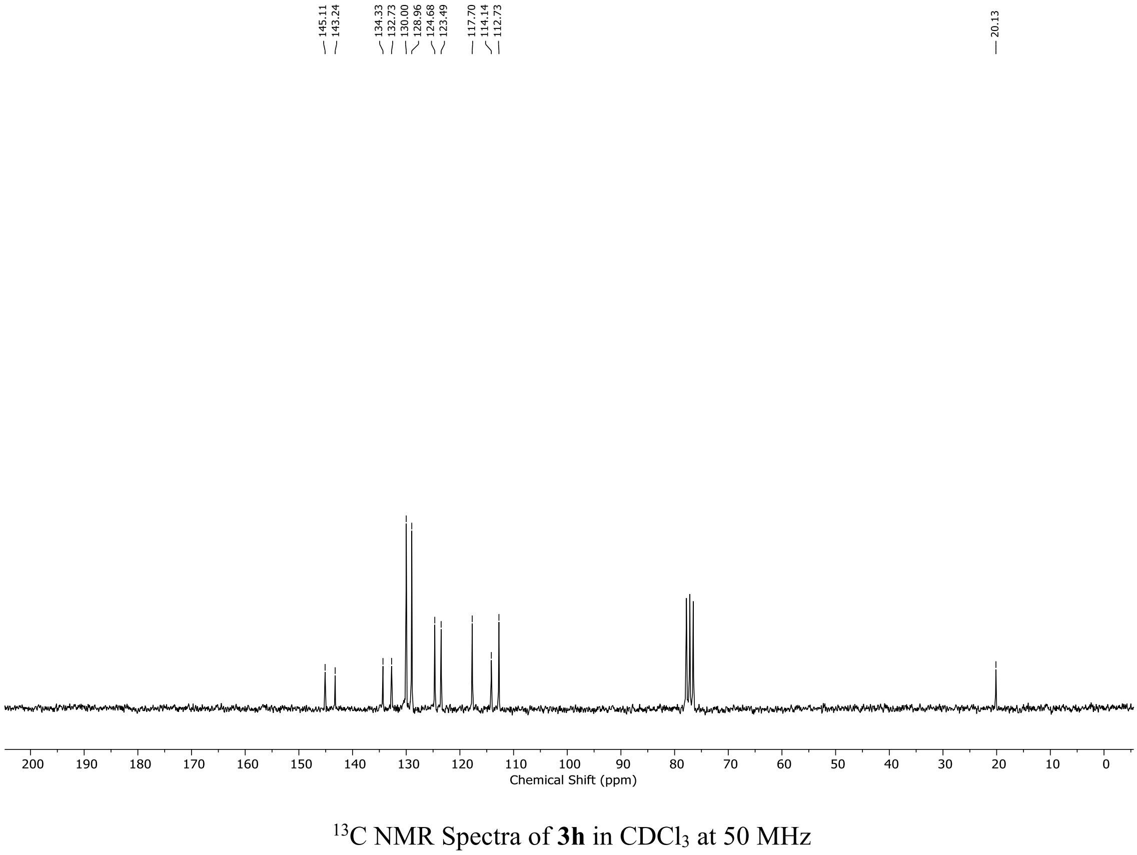

^{13}C NMR Spectra of **3h** in $CDCl_3$ at 50 MHz

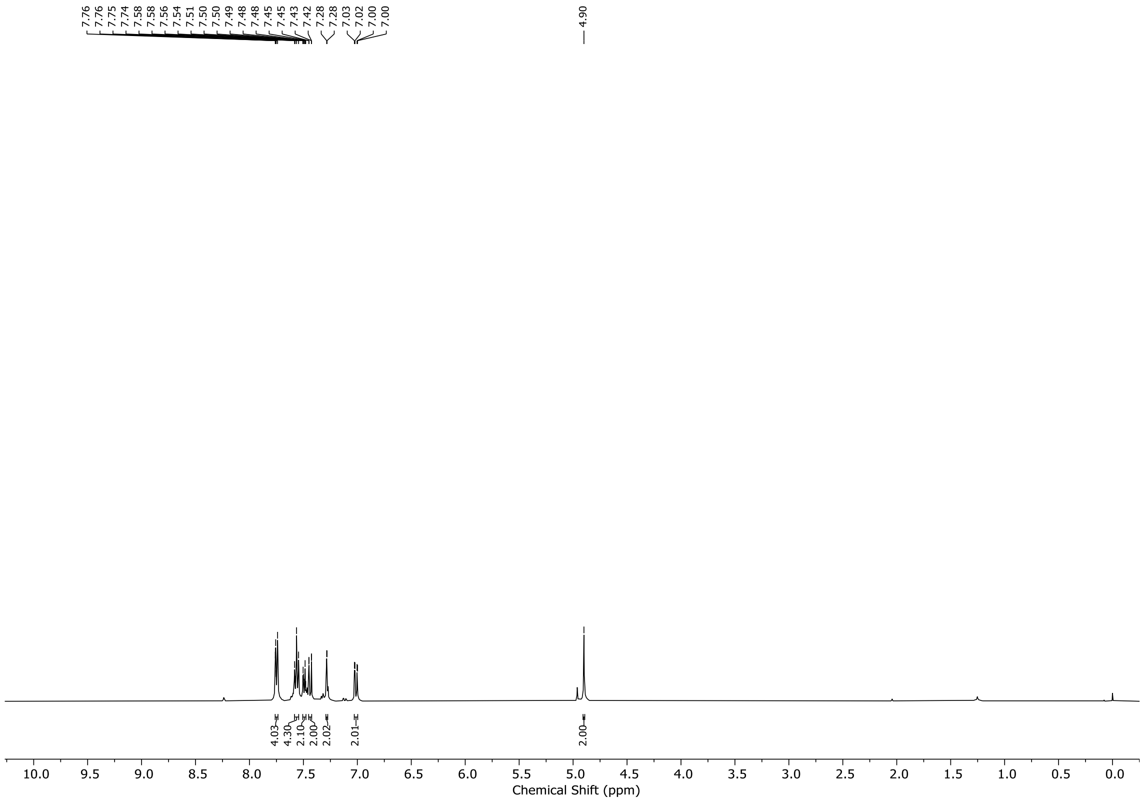

^1H NMR Spectra of **3i** in $CDCl_3$ at 400 MHz

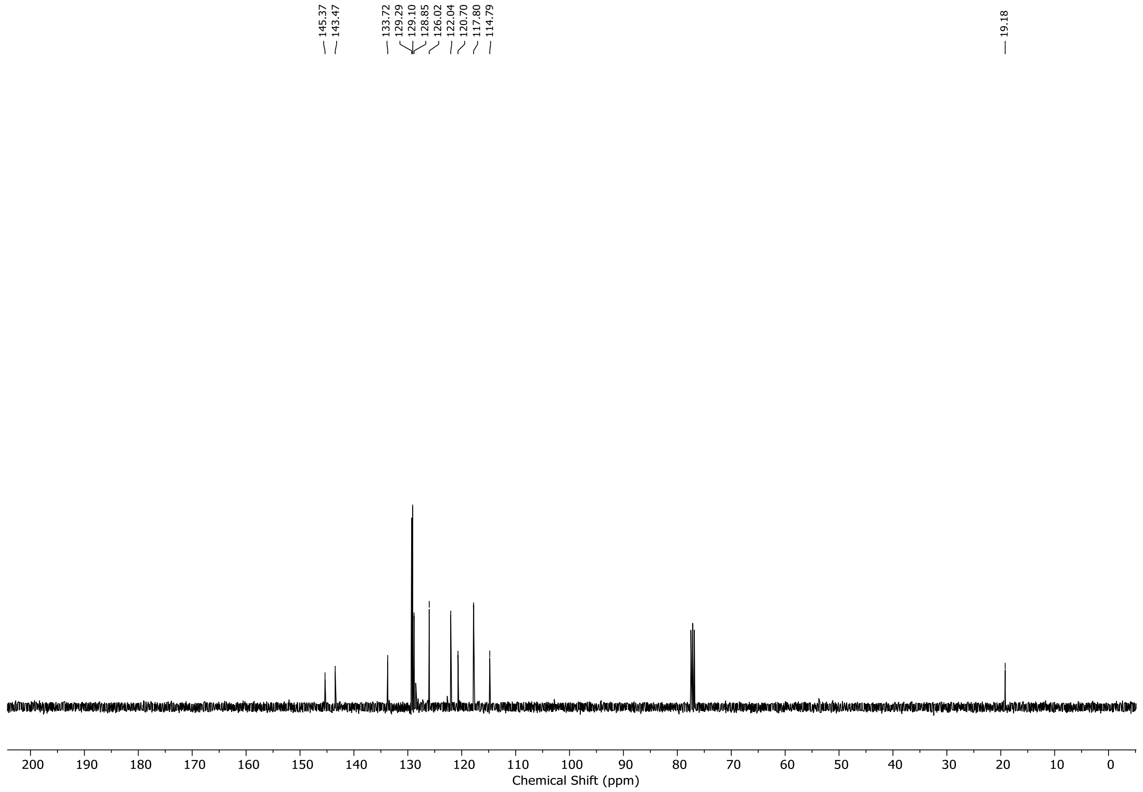

^{13}C NMR Spectra of **3i** in $CDCl_3$ at 100 MHz

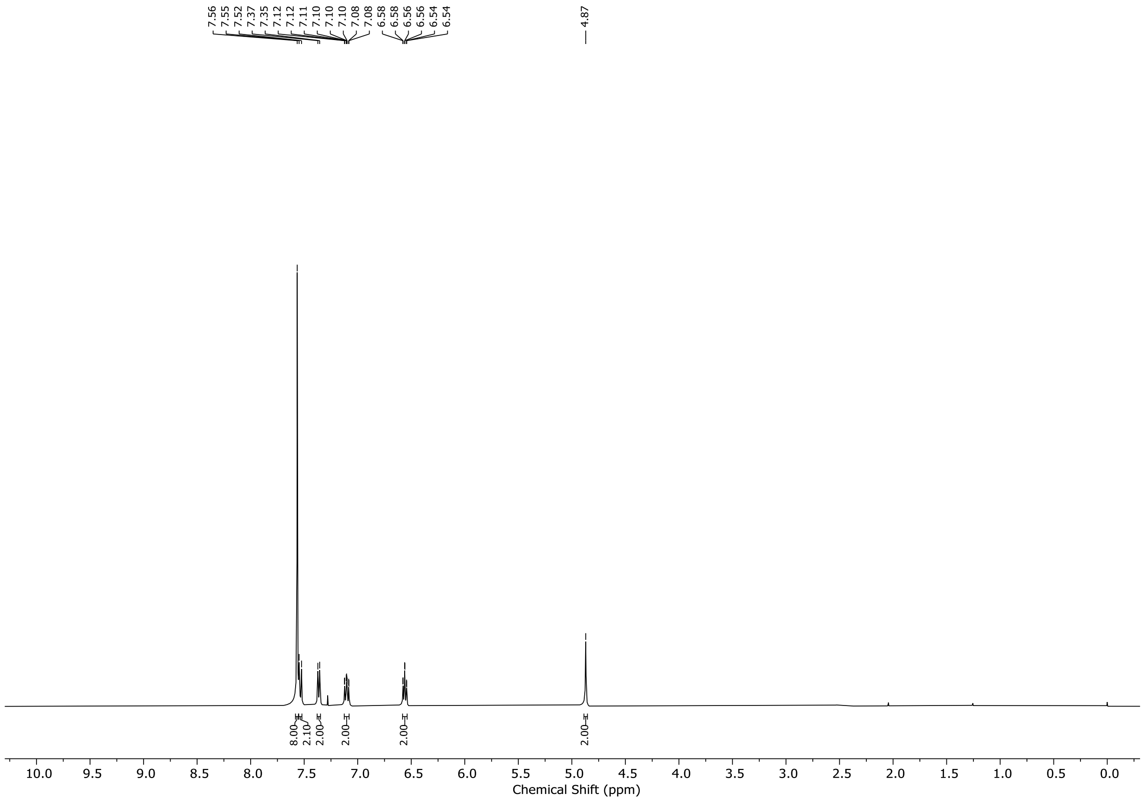

^{1}H NMR Spectra of **3j** in $CDCl_3$ at 400 MHz

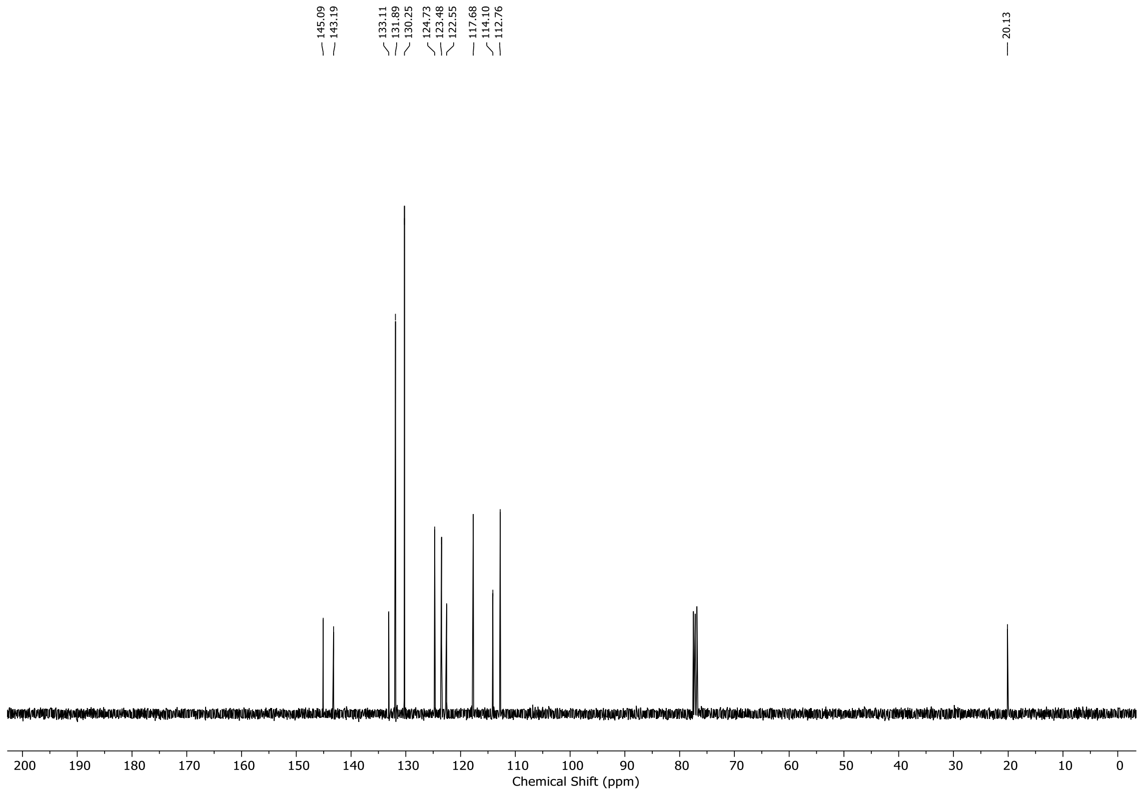

^{13}C NMR Spectra of **3j** in $CDCl_3$ at 100 MHz

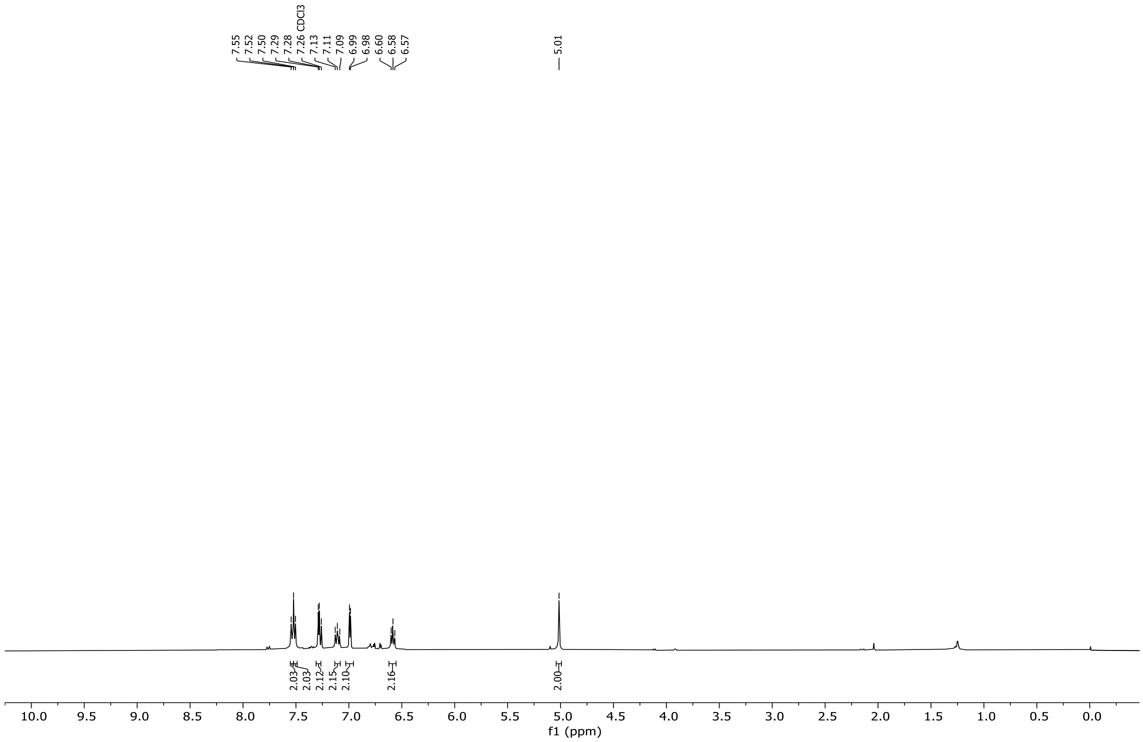

^{1}H NMR Spectra of **3k** in $CDCl_3$ at 400 MHz

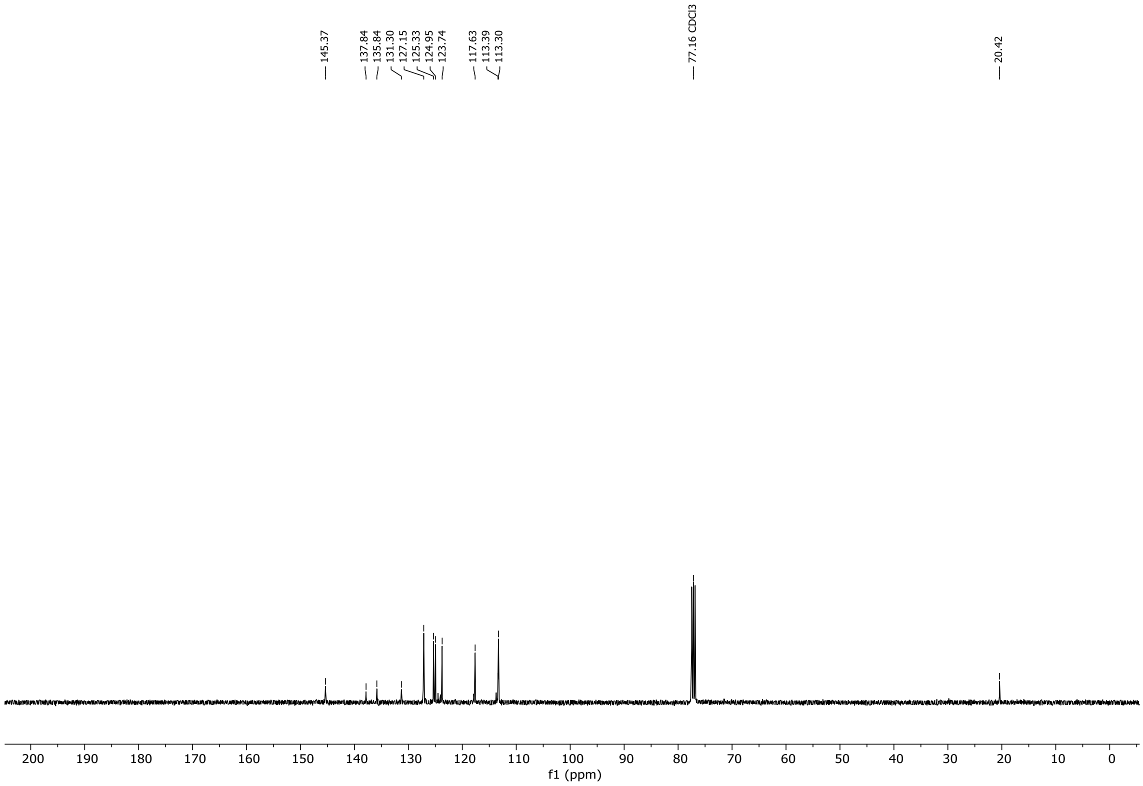

^{13}C NMR Spectra of **3k** in $CDCl_3$ at 100 MHz

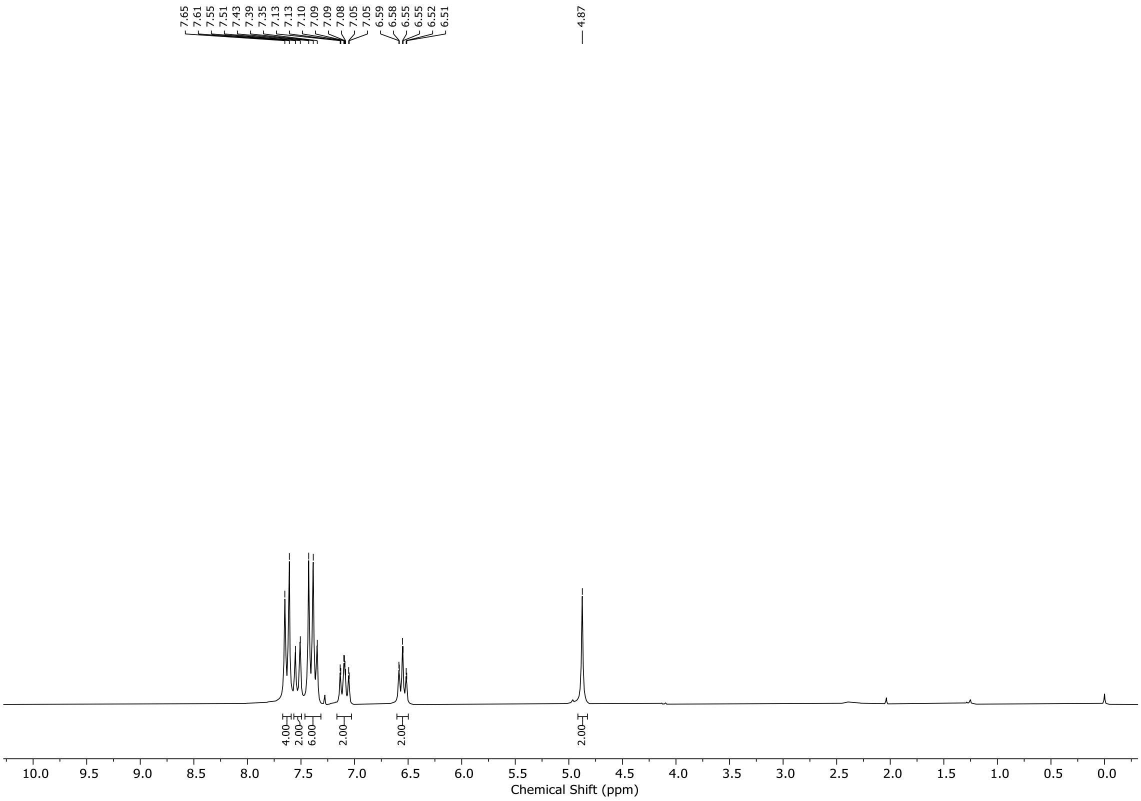

^{1}H NMR Spectra of **3l** in $CDCl_3$ at 200 MHz

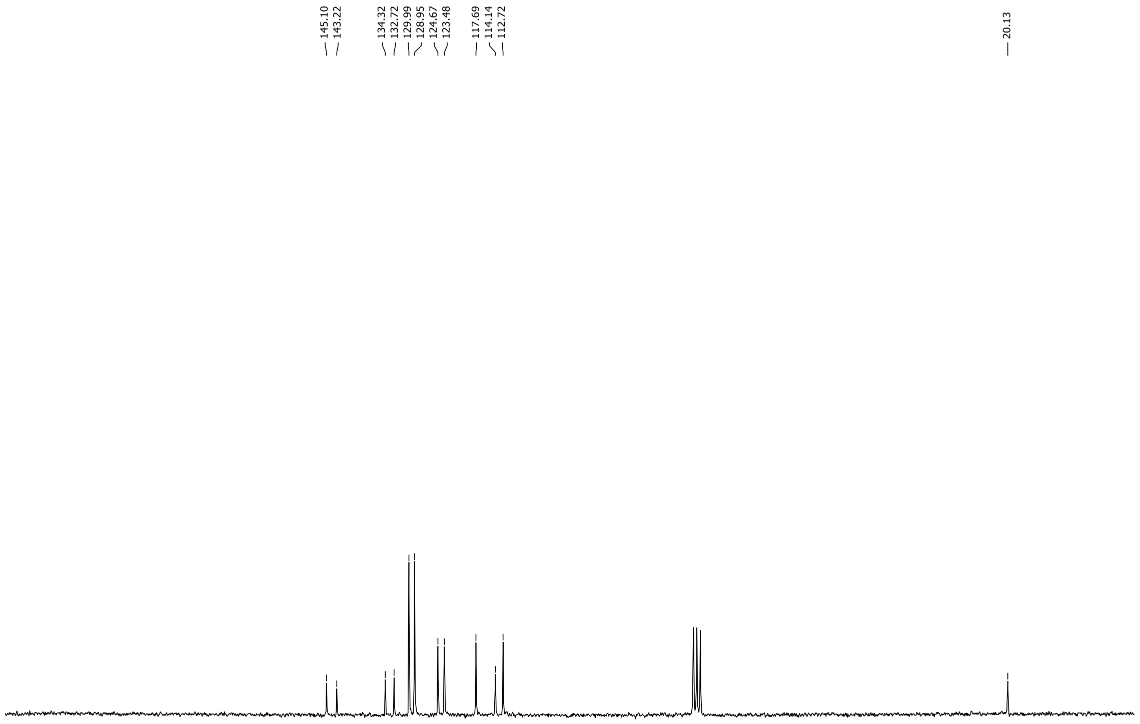

^{13}C NMR Spectra of **3l** in $CDCl_3$ at 50 MHz

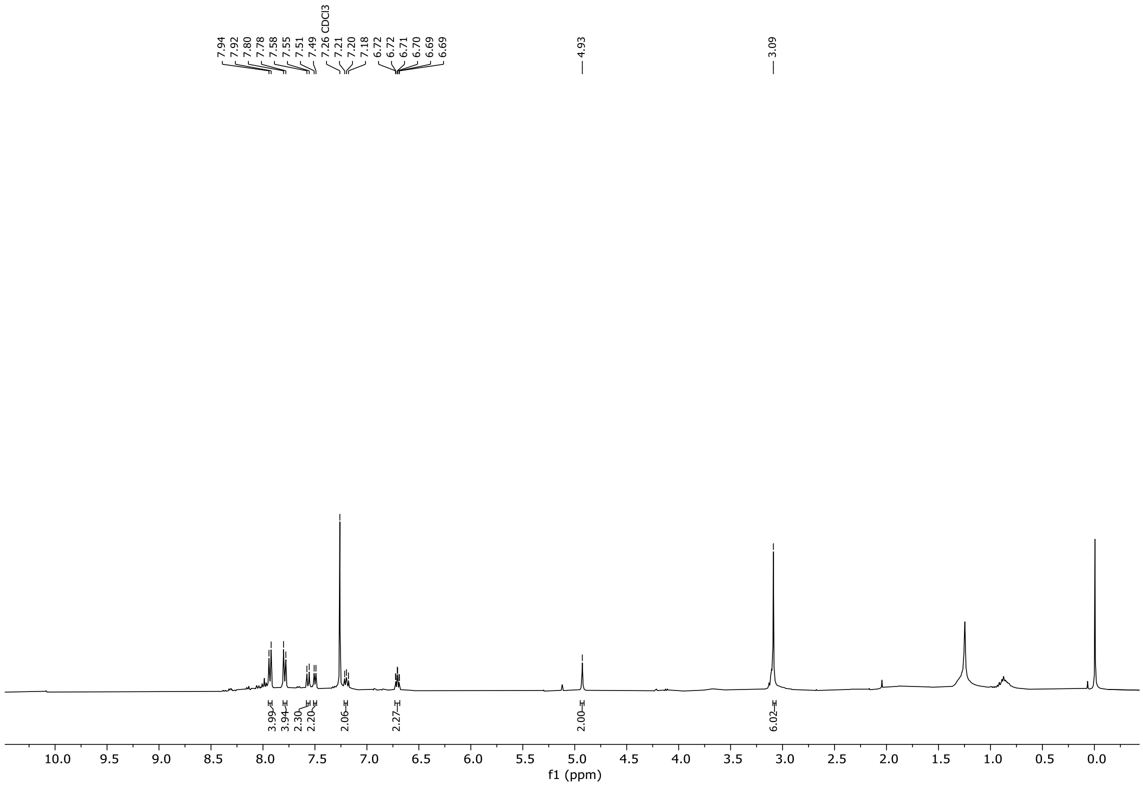

^{1}H NMR Spectra of **3m** in $CDCl_3$ at 400 MHz

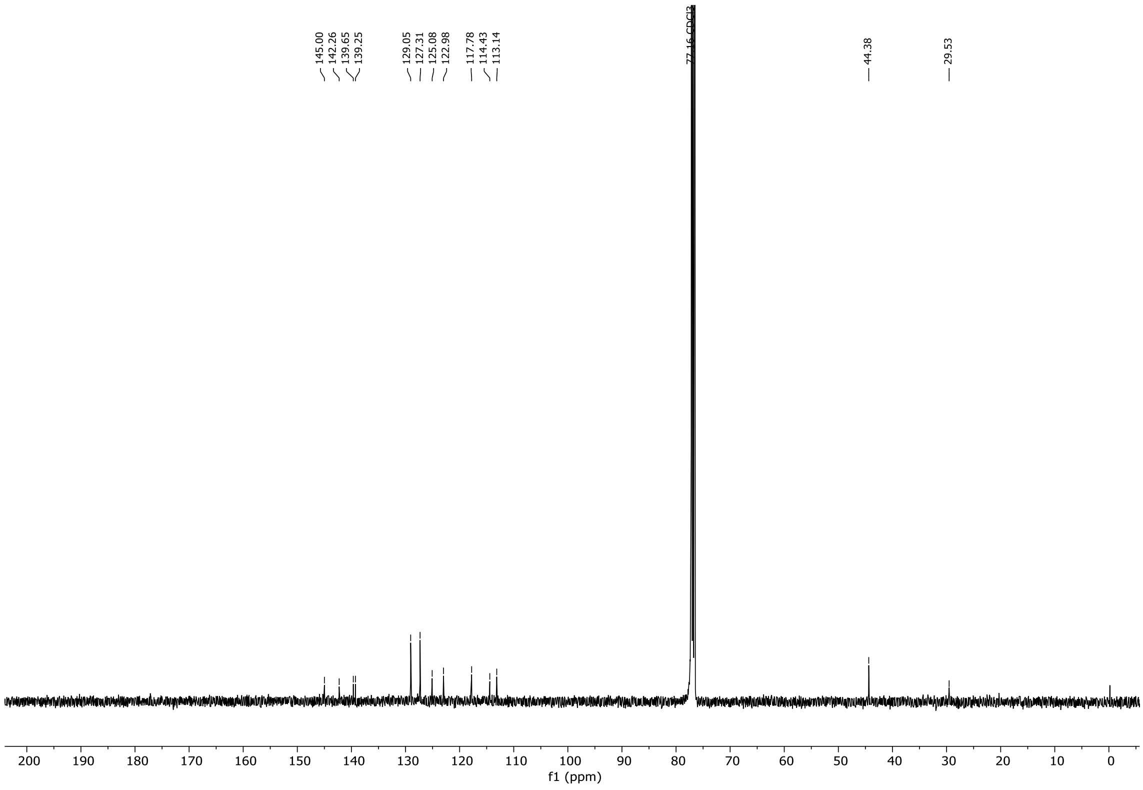

^{13}C NMR Spectra of **3m** in $CDCl_3$ at 100 MHz

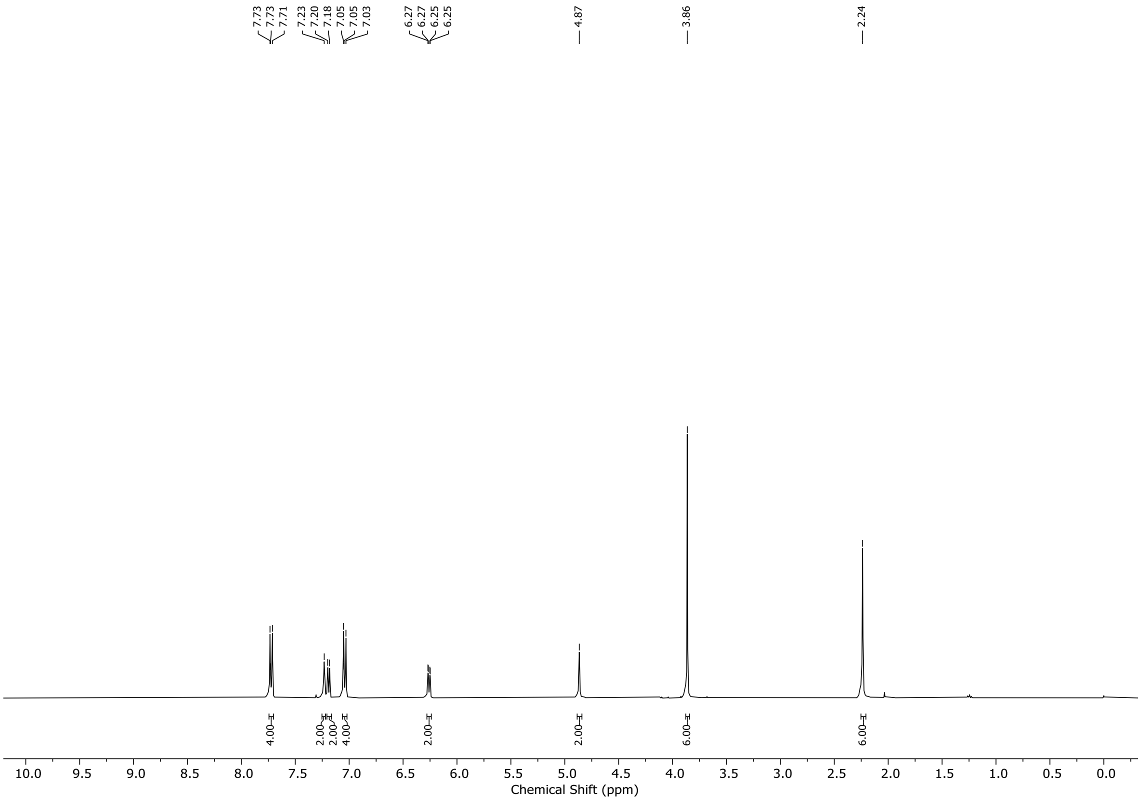

^{1}H NMR Spectra of **3n** in $CDCl_3$ at 400 MHz

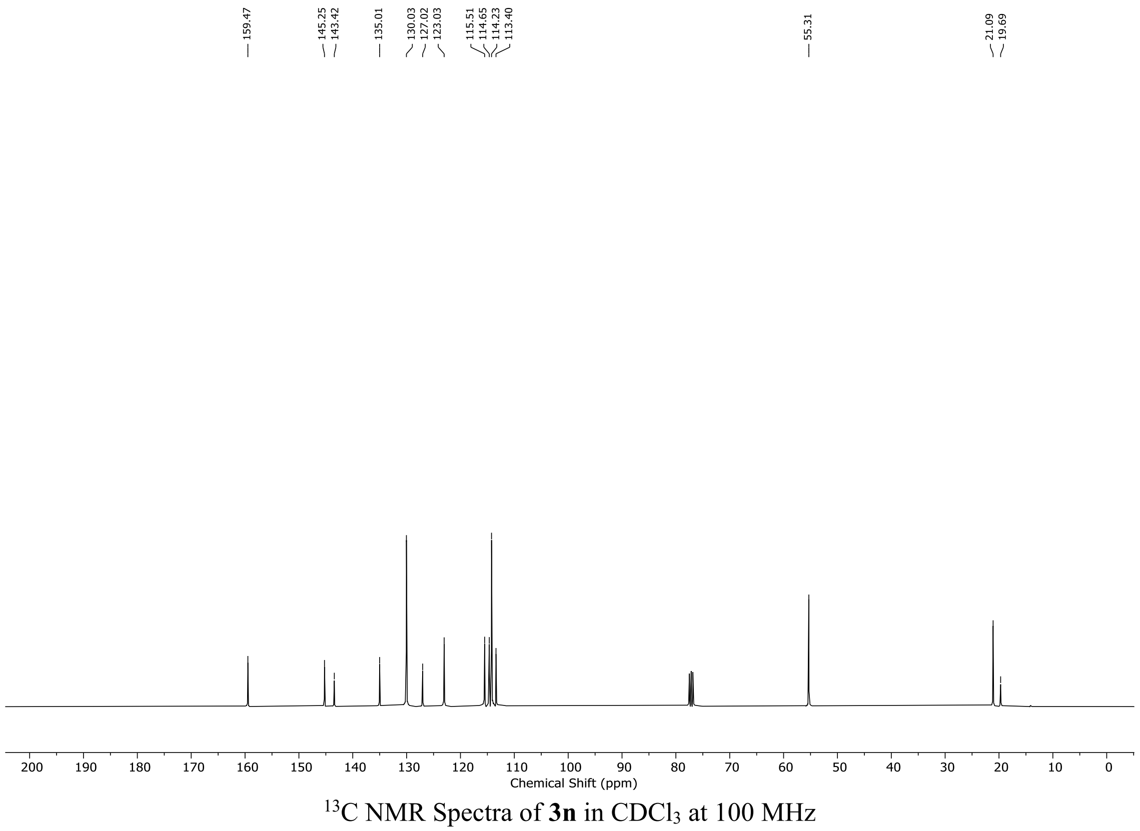

^{13}C NMR Spectra of **3n** in $CDCl_3$ at 100 MHz

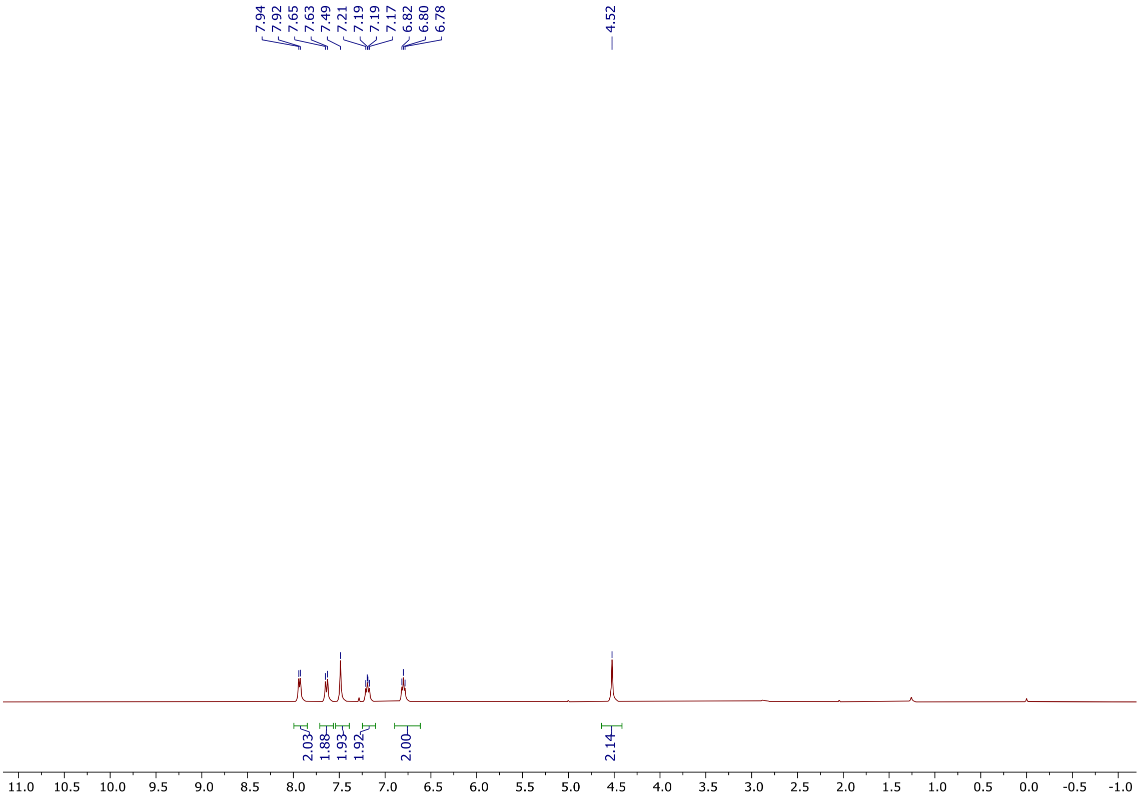

^{1}H NMR Spectra of **3o** in $CDCl_3$ at 400 MHz

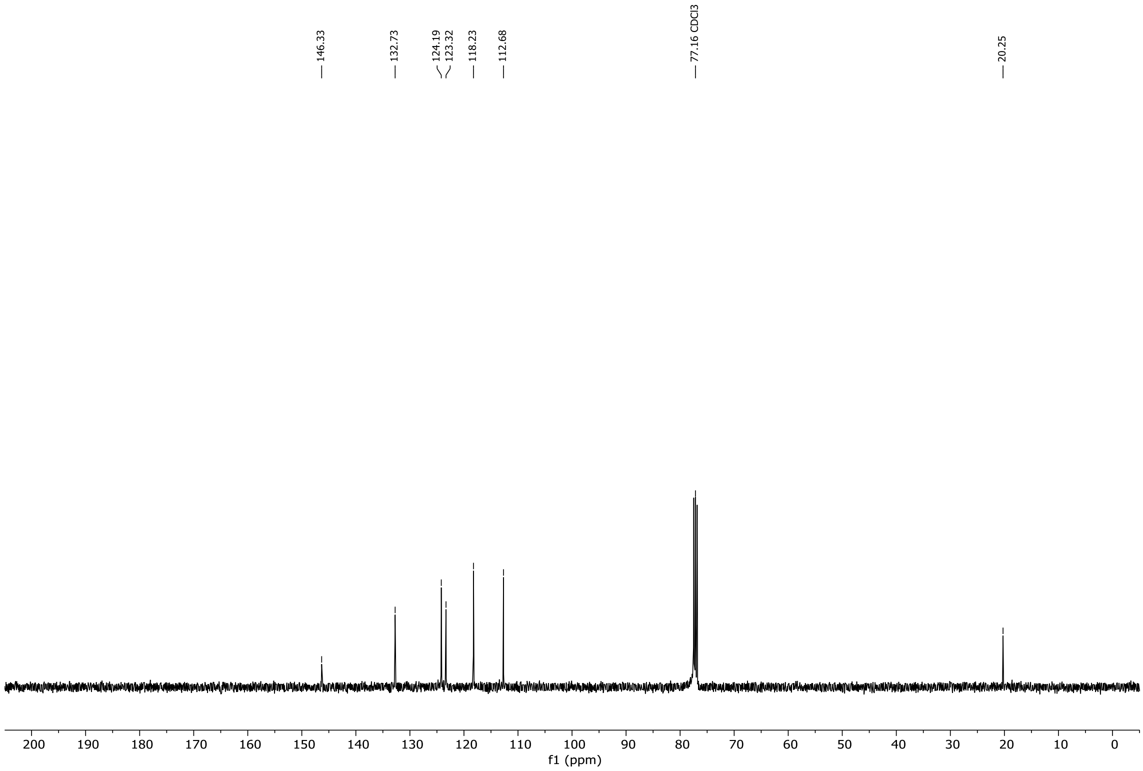

^{13}C NMR Spectra of **3o** in $CDCl_3$ at 100 MHz

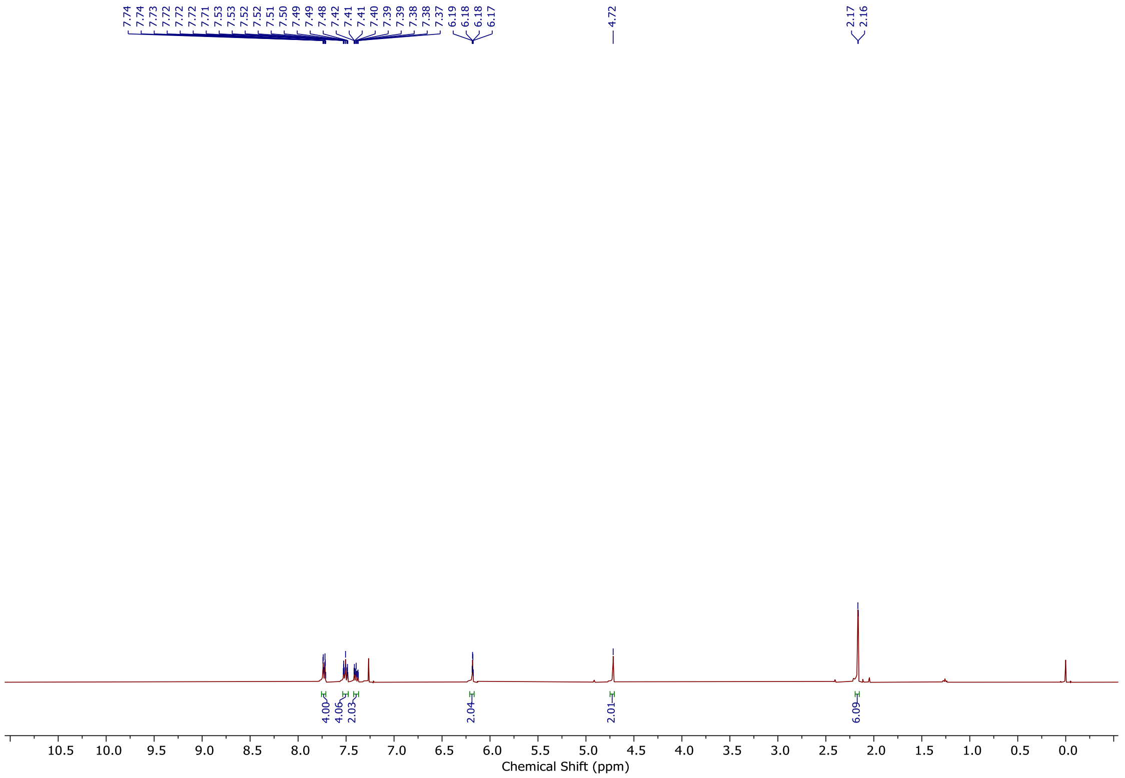

^{1}H NMR Spectra of **4a** in $CDCl_3$ at 400 MHz

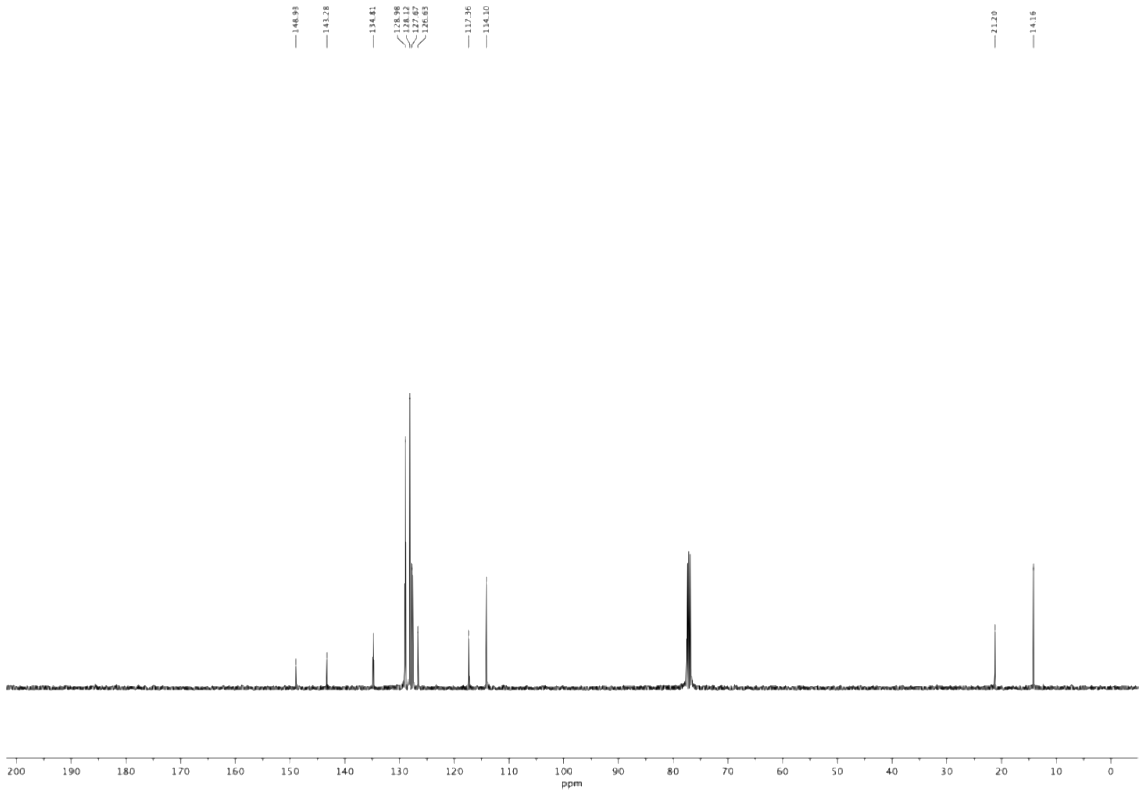

^{13}C NMR Spectra of **4a** in $CDCl_3$ at 100 MHz

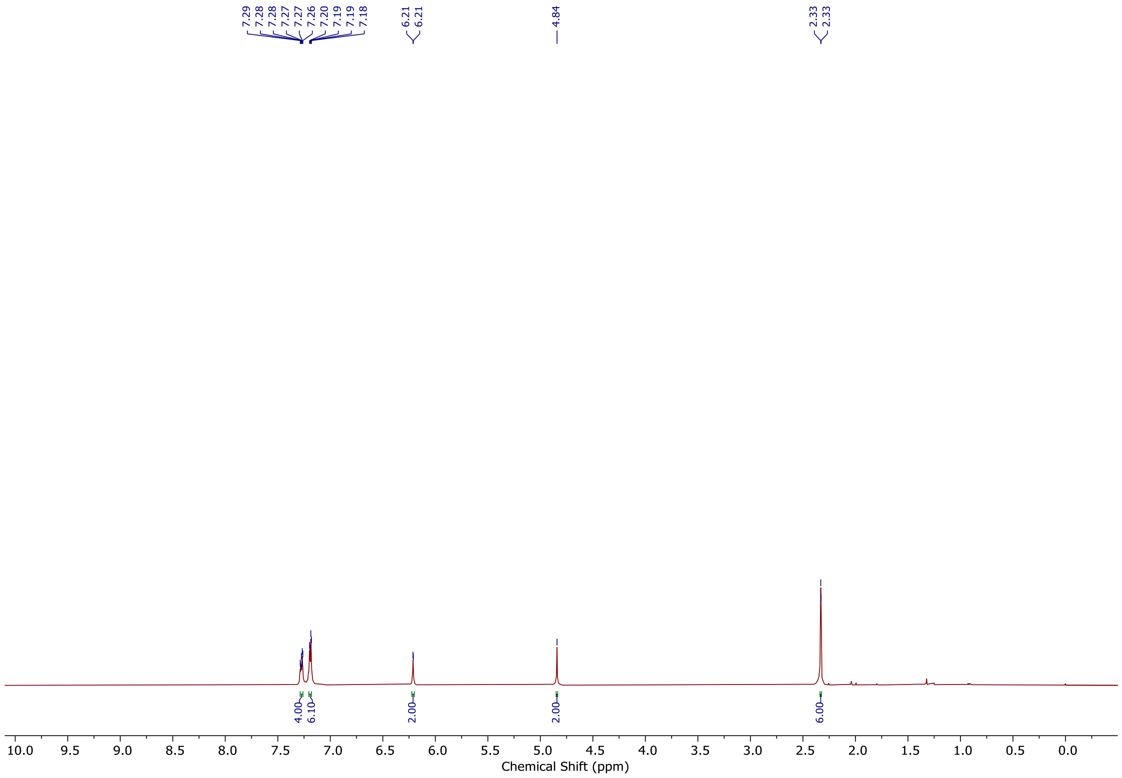

^{1}H NMR Spectra of **4b** in $CDCl_3$ at 400 MHz

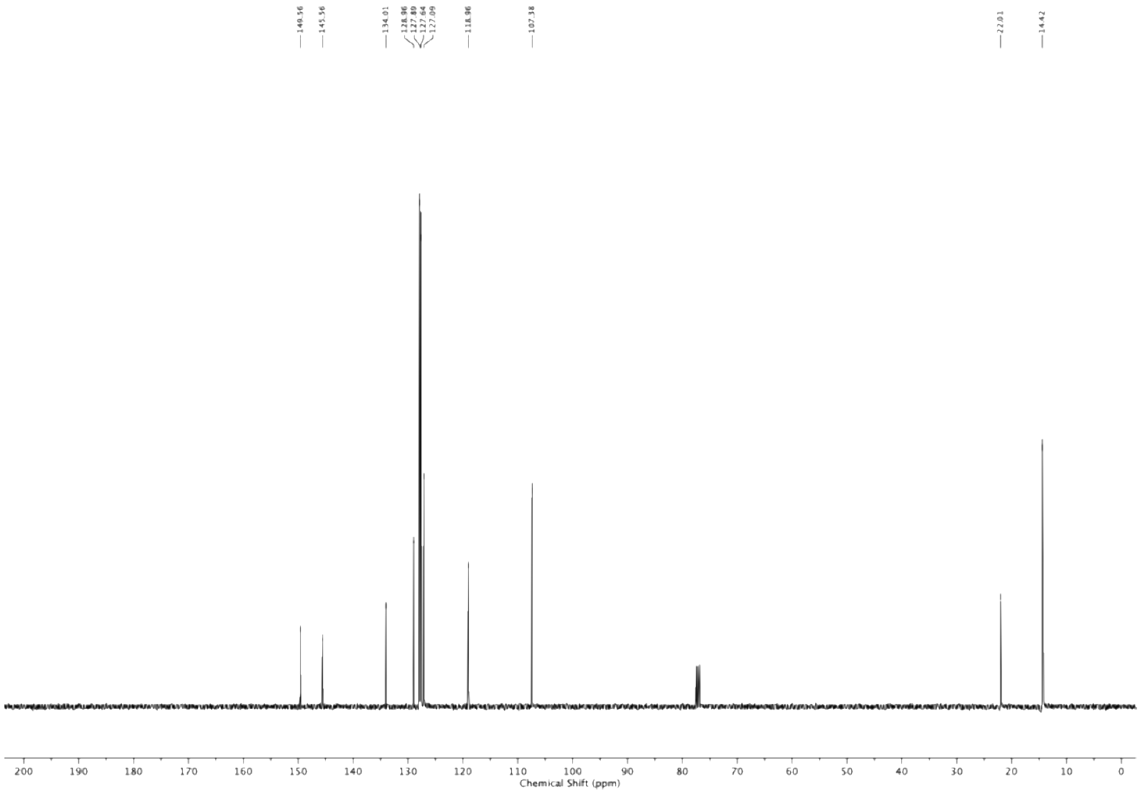

^{13}C NMR Spectra of **4b** in $CDCl_3$ at 100 MHz

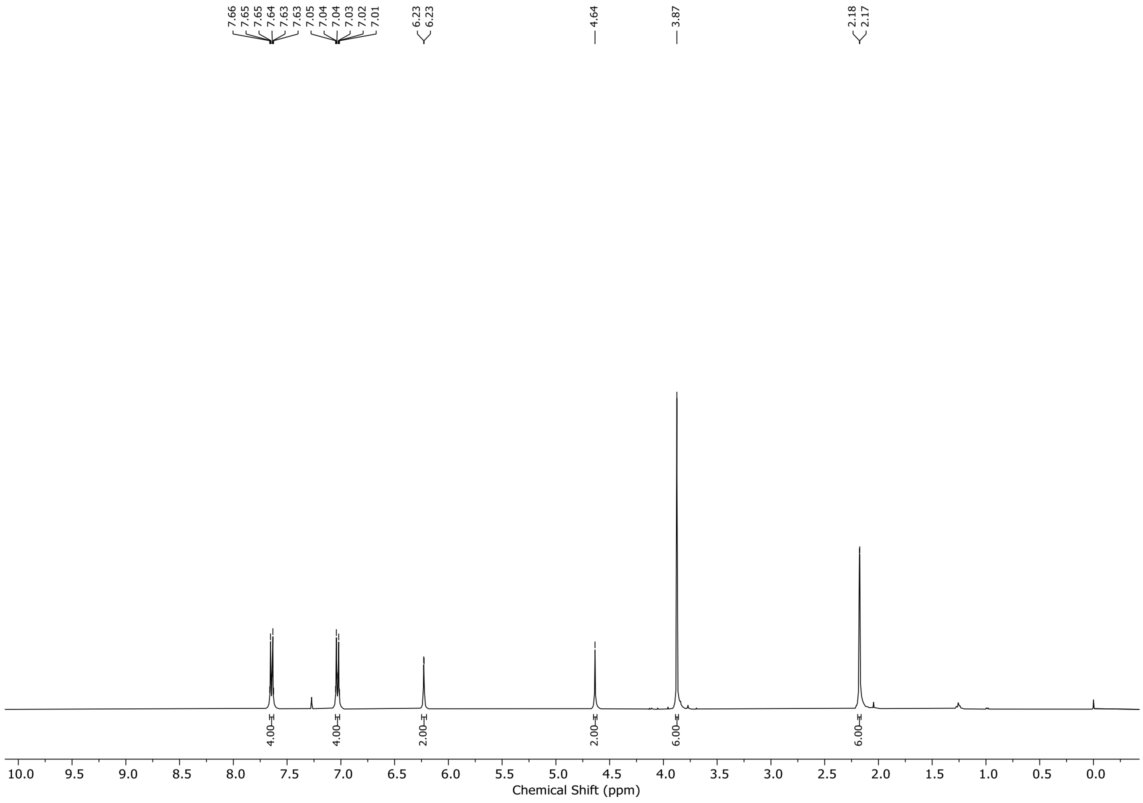

^{1}H NMR Spectra of **4c** in $CDCl_3$ at 400 MHz

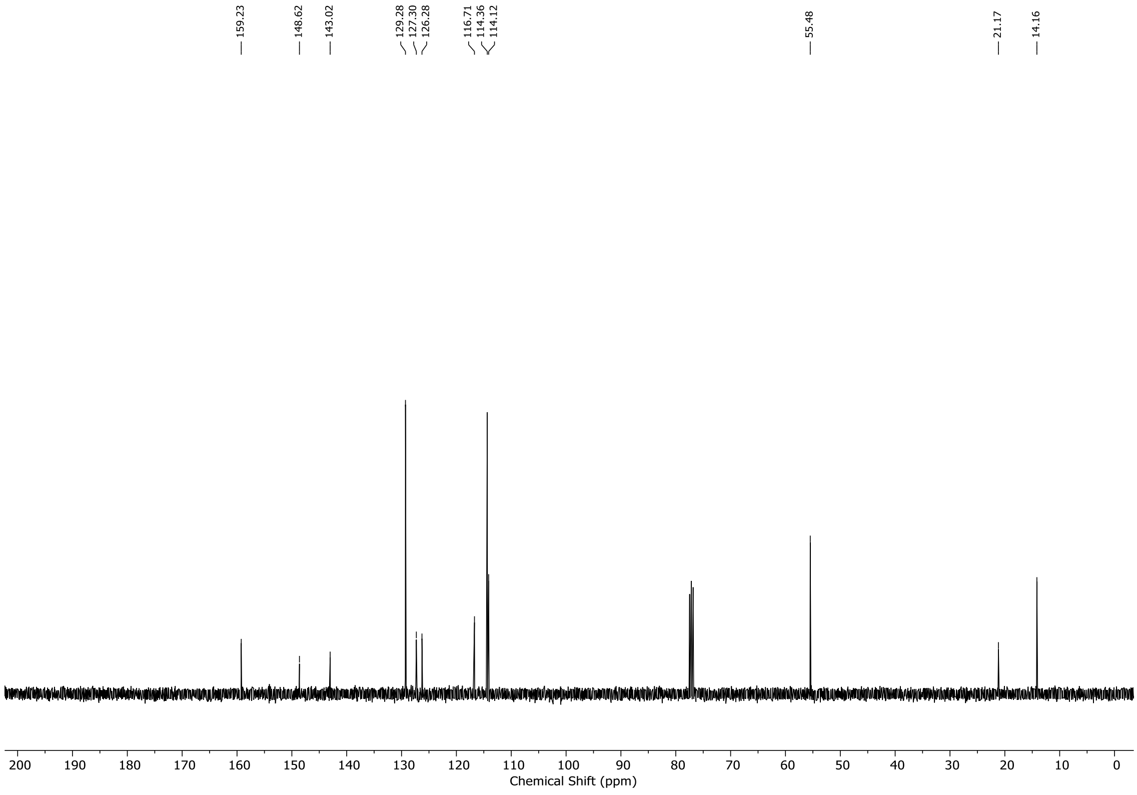

^{13}C NMR Spectra of **4c** in $CDCl_3$ at 100 MHz

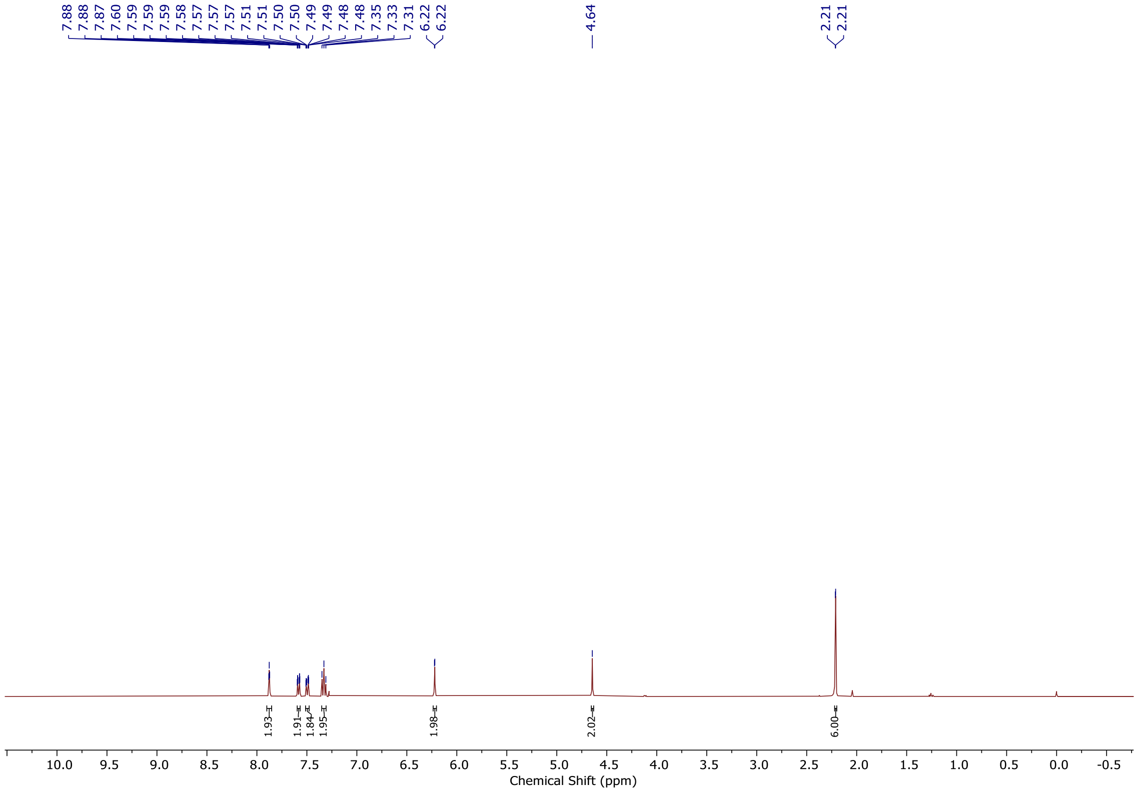

^{1}H NMR Spectra of **4d** in $CDCl_3$ at 400 MHz

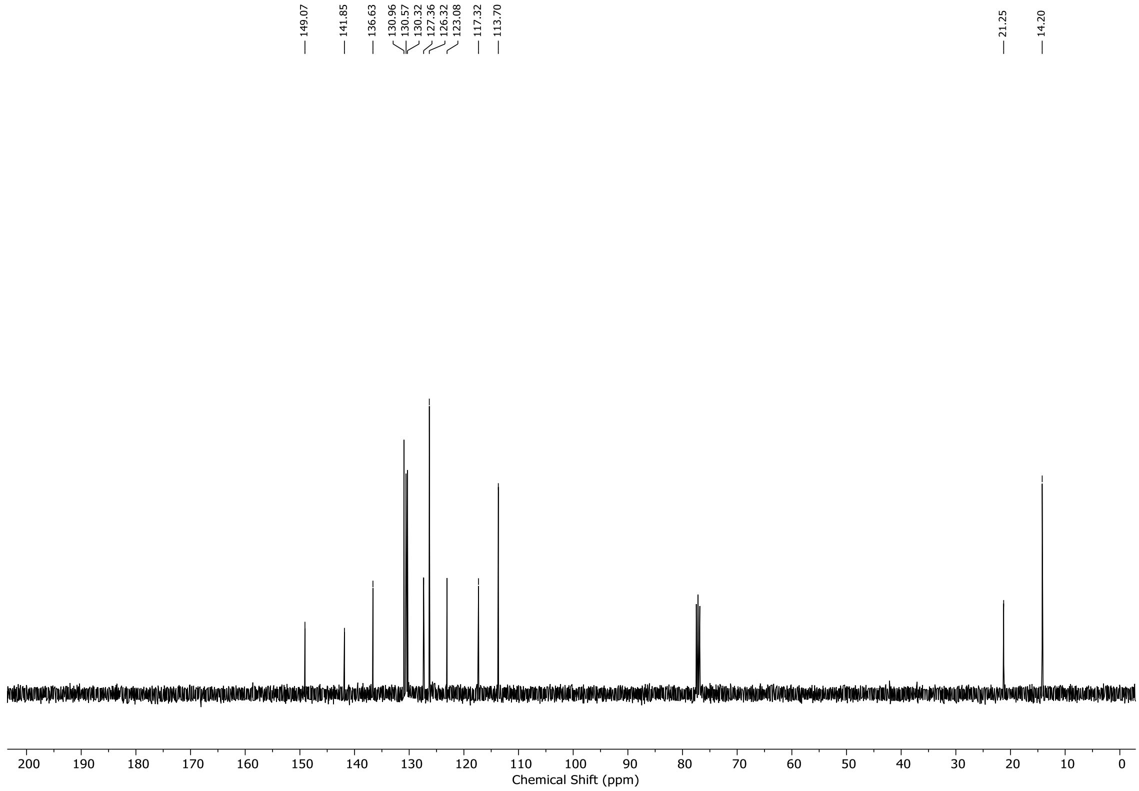

^{13}C NMR Spectra of **4d** in $CDCl_3$ at 100 MHz

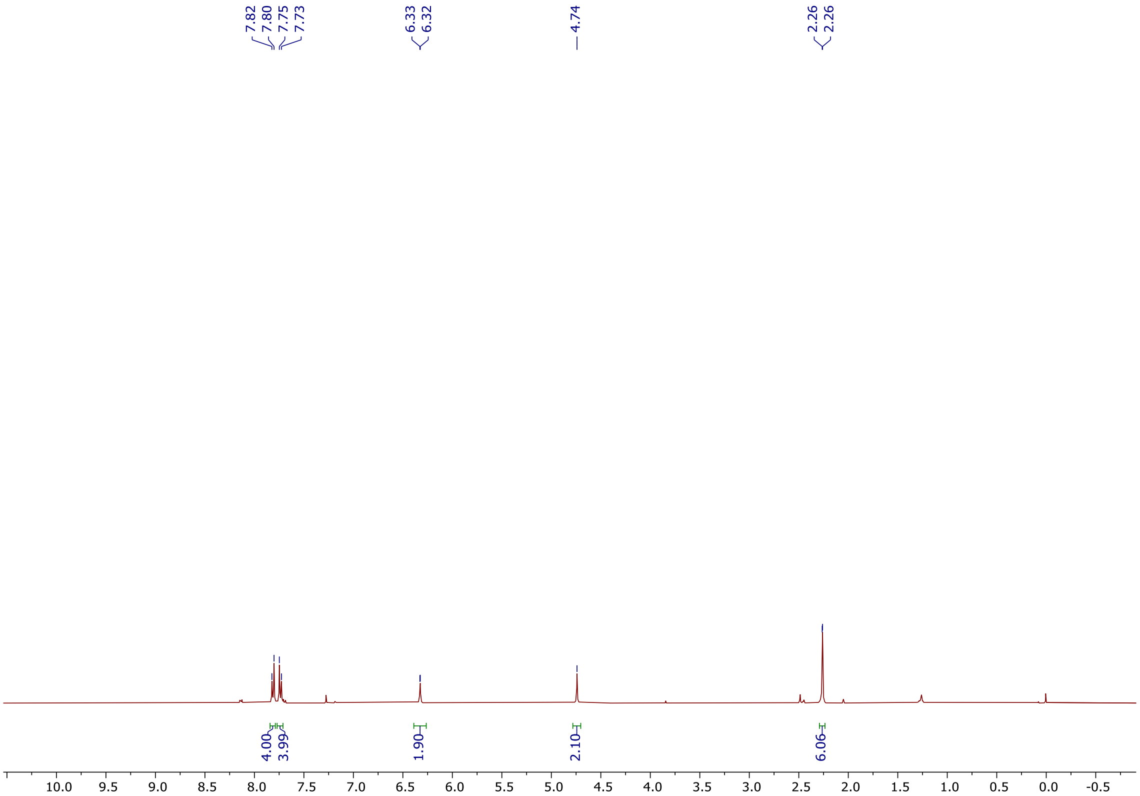

^{1}H NMR Spectra of **4e** in $CDCl_3$ at 400 MHz

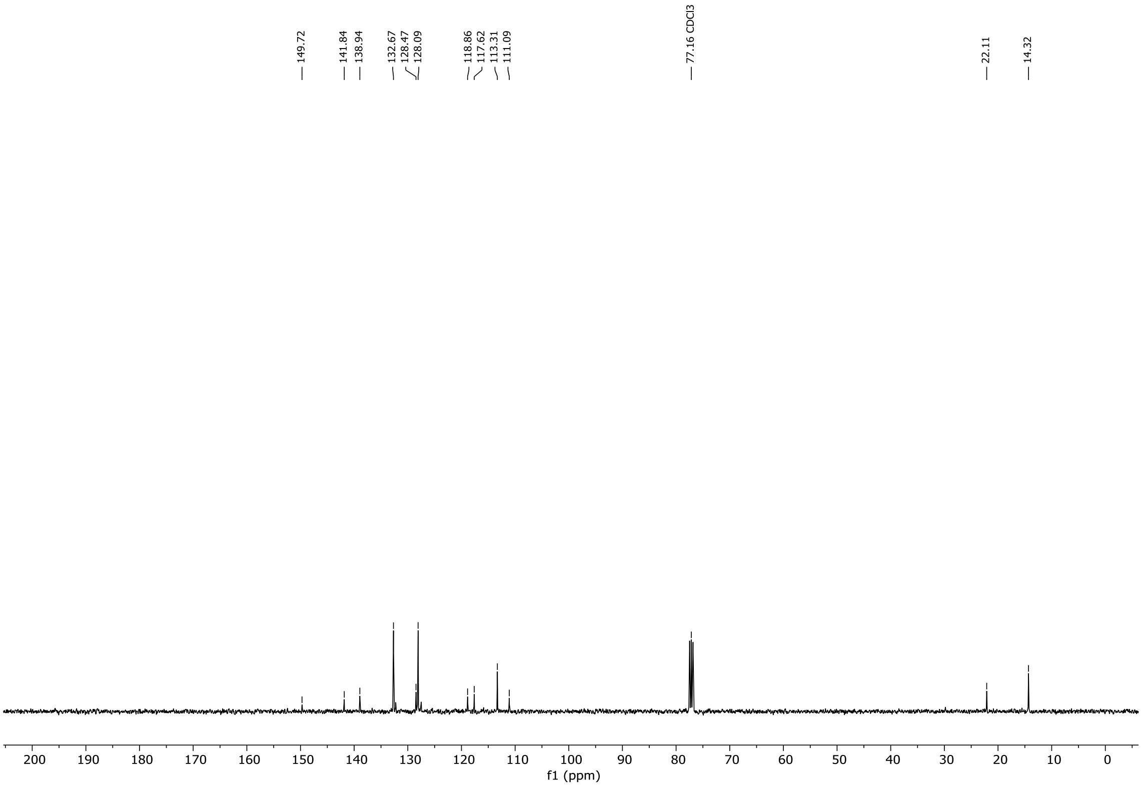

^{13}C NMR Spectra of **4e** in $CDCl_3$ at 100 MHz
